# Characterizing the temporal dynamics and maturation of brain activity during sleep: An EEG microstate study in preterm and full-term infants

**DOI:** 10.1162/imag_a_00450

**Published:** 2025-01-29

**Authors:** Parvaneh Adibpour, Hala Nasser, Amandine Pedoux, Laurie Devisscher, Nicolas Elbaz, Chloé Ghozland, Elodie Hinnekens, Sara Neumane, Claire Kabdebon, Aline Lefebvre, Anna Kaminska, Lucie Hertz-Pannier, Alice Heneau, Olivier Sibony, Marianne Alison, Catherine Delanoë, Richard Delorme, Marianne Barbu-Roth, Valérie Biran, Jessica Dubois

**Affiliations:** Université Paris Cité, INSERM, NeuroDiderot, Paris, France; Université Paris Saclay, CEA, NeuroSpin, UNIACT, Gif-sur-Yvette, France; Department of Physiology—Functional Explorations, Robert-Debré University Hospital, Assistance Publique-Hôpitaux de Paris—APHP, Paris, France; Department of Child and Adolescent Psychiatry, Robert-Debré University Hospital, APHP, Paris, France; Department of Pediatric Radiology, Robert-Debré University Hospital, APHP, Paris, France; Neonatal Intensive Care Unit, Robert-Debré University Hospital, APHP, Paris, France; Université Paris Cité, CNRS, Integrative Neuroscience and Cognition Center, Paris, France; Pediatric Physical Medicine and Rehabilitation Department, Raymond Poincaré University Hospital, APHP, Université Paris Saclay—Université Versailles St Quentin, Garches, France; Université Aix-Marseille, CNRS, Institute of Language, Communication and the Brain, Marseille, France; Department of Clinical Neurophysiology, Necker-Enfants Malades University Hospital, APHP, Paris, France; Department of Gynecology-Obstetrics, Robert-Debré University Hospital, APHP, Paris, France

**Keywords:** electroencephalography (EEG), resting-state, microstates, prematurity, brain development

## Abstract

By interfering with the normal sequence of mechanisms serving the brain maturation, premature birth and related stress can alter perinatal experiences, with potential long-term consequences on a child’s neurodevelopment. The early characterization of brain functioning and maturational changes is thus of critical interest in premature infants who are at high risk of atypical outcomes and could benefit from early diagnosis and dedicated interventions. Using high-density electroencephalography (HD-EEG), we recorded brain activity in extreme and very preterm infants at the equivalent age of pregnancy term (n = 43), and longitudinally 2 months later (n = 33), compared with full-term born infants (n = 14). We characterized the maturation of brain activity by using a dedicated microstate analysis to quantify the spatio-temporal dynamics of the spontaneous transient network activity while controlling for vigilance states. The comparison of premature and full-term infants first showed slower dynamics as well as altered spatio-temporal properties of brain activity in preterm infants. Maturation of functional networks between term-equivalent age and 2 months later in preterms was linked to the emergence of faster dynamics, manifested in part by shorter duration of microstates, as well as an evolution in the spatial organization of the dominant microstates. The inter-individual differences in the temporal dynamics of brain activity at term-equivalent age were further impacted by sex (with slower microstate dynamics in boys) and by gestational age at birth for some microstate dynamics but not by other considered risk factors. This study highlights the potential of the microstate approach to reveal maturational properties of the emerging brain network activity in premature infants.

## 1 Introduction

The last trimester of pregnancy is a critical phase of brain development, with vast growth of long-distance axonal connectivity, onset of myelination in white matter regions, rapid expansion of the cortex, synaptogenesis, and dendritic formation (Kostović & Judaš, 2015). Pre- and peri-natal insults such as preterm birth may impair the progression of these mechanisms, and potentially disrupt the trajectory of neurodevelopment, interfering with post-natal acquisitions and altering long-term cognitive, motor, and behavioral outcomes (Arpi & Ferrari, 2013;Bhutta et al., 2002). In particular, infants born before 32 weeks of gestational age (GA) are at a higher risk of adverse neurodevelopmental outcomes (Marlow et al., 2005;Pierrat et al., 2017,^2021^).

Magnetic Resonance Imaging (MRI) investigations have associated prematurity with alterations in the brain anatomical maturation by comparing preterm infants at term-equivalent age (TEA) with full-term neonates. These studies have described differences at the macro and microstructural scales, in cortical growth (Ajayi-Obe et al., 2000;Thompson et al., 2019), gyrification (Dubois et al., 2019;Engelhardt et al., 2015), microstructural organization (Bouyssi-Kobar et al., 2018;Dimitrova et al., 2021;Gondova et al., 2023), and white matter connectivity (Ball et al., 2014;Neumane et al., 2022), with differences that could persist into childhood (e.g.,Kelly et al., 2023a).

However, the functional correlates of such anatomical alterations are still poorly understood despite the common impairments observed at the behavioral level in children born preterm. In the past decade, functional MRI (fMRI) has allowed identifying precursors of spatially organized functional brain networks in the resting-state activity of preterms, and has shown reduced maturation in terms of long-range connectivity compared to full-terms (Doria et al., 2010) and alterations of network-level properties (Fenn-Moltu et al., 2023) that persist until TEA (Gozdas et al., 2018) and childhood (Wehrle et al., 2018). Yet, understanding how these alterations affect the efficiency of brain networks for processing environmental information requires investigating the fast dynamics of the brain activity that go beyond the temporal resolution of the Blood Oxygenation Level Dependent (BOLD) effect captured by fMRI.

While limited in spatial resolution, electroencephalography (EEG) can complement this picture by offering insights into the fast dynamics of the brain activity and by being easily implementable in vulnerable infants. Previous EEG investigations of preterms have indeed identified differences in the occurrence of discontinuous high-amplitude bursts (Whitehead et al., 2017), activity synchronization (Yrjölä et al., 2022), spectral density (Guyer et al., 2019), and in the latency (Kaminska et al., 2018) and scalp distribution of sensory-evoked responses (Maître et al., 2017;Whitehead et al., 2019), with aspects of these alterations persisting into TEA and beyond (Guyer et al., 2019). These investigations indicate that several dimensions of the brain activity such as temporal, scalp distribution, amplitude, or signal strength can be impacted by prematurity. Moreover, when investigating the brain spontaneous activity, most methods (i.e., spectral analysis, functional connectivity) study the brain dynamics over several seconds, limiting their interpretation of the temporal properties at a finer scale. Similarly, EEG clinical evaluations involve assessments of sleep structure and maturational figures over relatively long timescales (Dereymaeker et al., 2017), while the brain activity experiences fast changes in its functional organization (Baker et al., 2014), supporting development and learning during sleep (Li et al., 2017). These encourage the use of methods that capture multidimensional information about the brain activity while being sensitive to the fine temporal aspects of the brain dynamics, in order to provide reliable markers of functional alterations in preterm infants.

Microstate analysis aims at identifying short time-segments within the continuous recordings, where the scalp topography (i.e., the distribution of electric potentials originating from the brain activity over the scalp) remains quasi-stable before transitioning into a new scalp topography (Lehmann et al., 1987;Michel & Koenig, 2018). Each of these transient topographies is defined as a microstate (MS), representing the spontaneously occurring transient network activity. Microstates are, thus, sensitive to both temporal and scalp topographical dynamics of the brain activity.

Recently, the interest in using the microstate approach in infants has emerged, while eliciting various methodological questions (Bagdasarov et al., 2024;Brown & Gartstein, 2023;Khazaei et al., 2021). But studies in neonates are rare, focusing on the impact of sleep states on brain dynamics (Khazaei et al., 2021) and on habituation mechanisms to pain (Rupawala et al., 2023), with recent reports indicating microstate dynamics change in the preterm period toward the term-age (Hermans et al., 2024). Yet, the period of the first months after term birth remains unexplored with the microstate approach so far, whereas the acceleration of brain functional dynamics and the evolution of network activity topographies are expected to result from the intense progression of maturational processes (e.g., myelination and growth of short cortico-cortical connections) (Kostović & Judaš, 2015). High-density EEG (HD-EEG) recordings might then benefit the characterization of spatio-temporal properties of the brain activity, to better reflect the anatomo-functional changes in the early developmental period. In addition to investigating brain dynamics in the first months after term birth, the potential impact of perinatal risk factors such as prematurity on microstate-related characteristics still needs to be understood. This is of particular importance, given the early onset and heterogeneity of cerebral atypicalities (Dimitrova et al., 2020), requiring the investigation of developmental trajectories through longitudinal assessments.

In this study, we thus aimed to characterize the functional maturation of brain activity dynamics in high-risk preterm infants using HD-EEG recordings. We used the microstate approach to study the maturation of neurophysiological activity between two time-points during development: at term-equivalent age and 2 months later. First, we compared preterm and full-term born infants in order to identify how the spatio-temporal properties of brain dynamics are impacted by prematurity, while controlling for vigilance states and focusing on sleep states in the main analyses. Next, we implemented a dedicated approach to reliably characterize and compare the organization of spontaneous brain activity in preterms across the two ages. Then, we evaluated how the metrics describing the dynamics of such a functional organization mature over 2 months after TEA, and we investigated to which extent the inter-individual variability in these dynamics is related to perinatal clinical factors. We hypothesized that spatio-temporal properties of microstates can capture functional aspects of brain activity maturation, which may be sensitive to prematurity and its related risk factors. In particular, we expected an acceleration of the temporal features describing microstates with maturation, which, in turn, predicted: 1) longer microstate duration (i.e., slower brain dynamics) in preterms vs. full-terms, 2) longer microstate duration in infants with higher degrees of perinatal risk factors, and 3) shortening of microstate duration from the term-equivalent age to 2 months later. Since asynchronous maturation of the different brain networks creates distinct time windows of vulnerability across cerebral networks (Neumane et al., 2022;Wu et al., 2017), we did not expect all microstates to be impacted similarly by prematurity.

## 2 Methods

The study design and procedures (protocol DEVine, CEA 100 054) was reviewed and approved by the ethics committee (Comité de Protection des Personnes, CPP Ile de France 3). Parents were informed about the study and gave written consent to participate with their babies. The study involved newborns admitted to the Robert-Debré Children’s Hospital (Paris, France), who were born preterm and followed in the Neonatal Intensive Care Unit, or born full-term in the Maternity.

### 2.1 Participants

This study considered preterm infants born before 32 w GA who were due to have a clinical MRI exam at TEA (because of GA lower than 28 w or clinical/neurological suspicions) and HD-EEG recordings for research purposes. The MRI exam (obtained in all except one infant) allowed us to measure the Kidokoro score, summarizing the brain development and regional abnormalities (Kidokoro et al., 2013). All infants except one showed a Kidokoro total score (for the whole brain) that was considered as “normal” (score ≤ 3) or related to “mild” abnormalities (4 ≤ score < 8). To favor a relatively homogeneous group, these two infants with no MRI or moderate/severe brain injury were not included in this EEG study.

The preterm cohort then included a group of 43 extreme (born before 28 weeks) and very preterm (born between 28 and 32 weeks) infants (mean GA at birth: 27.1 ± 1.7 weeks, range: [24.1–0.9] weeks), as defined by the World Health Organization guidelines (World Health Organization, 2023). Infants were tested longitudinally at two time points: first, at the term-equivalent age (0 months Corrected Age – 0 mCA: 41 ± 0.8 weeks of post-menstrual age – w PMA, range: [38.4–42.3] weeks; 20 females, 23 males), and next at 2 months corrected age (2 mCA: 50.5 ± 0.7 w PMA, range: [49.1–52] weeks; 16 females, 17 males, leading to 33 infants at 2 mCA since 10 did not return for the second exam).

Table 1summarizes the neonatal characteristics of the preterm infants (seeSupplementary Table 1for the details of individual characteristics). Based on previous studies, we selected and collected data on the clinical factors supposed to play a key role on the neurodevelopment of premature neonates and known as risk factors of adverse outcomes, including GA at birth (considering three GA groups: GA1: 24 weeks + 0 day ≤ GA ≤ 26 weeks + 0 day/GA2: 26 weeks + 1 day ≤ GA ≤ 28 weeks + 0 day/GA3: 28 weeks + 1 day ≤ GA ≤ 32 weeks + 0 day), sex, birth weight (correcting for GA and considering values below the 10^th^percentile as small weight for GA), as well as non-neurological complications during the NICU period: chronic lung disease, need for invasive mechanical ventilation (oxygen therapy) lasting strictly more than 1 day, need for parenteral nutrition longer than 3 weeks, necrotizing enterocolitis, and experiencing sepsis (Brouwer et al., 2017;Neumane et al., 2022). Necrotizing enterocolitis was confirmed by the neonatal clinical team and defined as the presence of clinical evidence meeting the modified criteria for NEC Bell’s stage II, associated with radiologic pneumatosis intestinalis, or stage III determined as definitive intestinal necrosis seen at surgery or autopsy (Walsh & Kliegman, 1986). Sepsis was identified in early and late onset cases on the basis of positive blood culture and/or cerebrospinal fluid culture to a pathogen (Sikias et al., 2023;US Centers for Disease Control and Prevention, 2024;Wynn et al., 2014). From these last 5 clinical factors of non-neurological complications, we derived 1) a binary factor of neonatal morbidities associated with prematurity (as inNeumane et al., 2022), to summarize the presence of at least one of them (or the absence of all five), and 2) a continuous ratio score ranging from 0 (no clinical factor) to 1 (all 5 factors) and with 0.2 steps, to summarize how many of the five factors were present in each infant.

**Table 1.** Summary of neonatal characteristics of the infants at birth.

Subjects	n = 43
GA at birth	27.1±1.7 w Min = 24.1 w Max = 30.9 w
GA category	n = 13/19/11 GA1/GA2/GA3
Sex	n = 23/20 M/F
Small weight for GA	n = 8/35 Yes/No
MRI Kidokoro score	n = 7/36 Mild/Normal
Chronic lung disease	n = 25/18 Yes/No
Mechanical ventilation > 1 day	n = 17/26 Yes/No
Necrotizing enterocolitis	n = 7/36 Yes/No
Parenteral nutrition > 3 weeks	n = 20/23 Yes/No
Sepsis	n = 32/11 Yes/No
Neonatal morbidity risk	n = 37/6 Yes/No

Numbers of infants are described over groups of GA at birth (GA1/GA2/GA3), sex (M/F), binarized risk of birth weight indicating small for gestational age (yes/no), binarized MRI Kidokoro score (mild/normal), as well as information regarding five categories of non-neurological complications at NICU. These complications were considered for chronic lung disease (yes/no), use of invasive mechanical ventilation for more than 1 day (yes/no), necrotizing enterocolitis (yes/no), parenteral nutrition for more than 3 weeks (yes/no), and sepsis (yes/no), which were then summarized as one single binarized score of neonatal morbidity factor (yes: at least one of the five identified non-neurological complications/no: none). SeeSupplementary Table 1for the report of individual characteristics.

The full-term cohort included a group of 14 infants (mean GA at birth: 40.1 ± 1.1 weeks, range: [37.6–41.6] weeks) who were tested first a few days/weeks after birth (mean age at test: 41.6 ± 1.1 w PMA, range: [39.7–43.3] weeks; 7 females, 7 males) and 2 months later (mean age at test: 50.1 ± 0.9 w PMA, range: [48.9–51.9] weeks; 7 females, 6 males, leading to 13 infants at 2 mCA since 1 infant did not return for the second exam).

### 2.2 EEG recording

High-density 128-channel electroencephalography (HD-EEG, Magstim EGI, Eugene, USA) was recorded on average for approximately 10 minutes during rest, while infants were staying calm in a baby cot, or occasionally in the parent’s arms when it was easier to keep the infant rested. Sampling rate of the recordings was 1,000 Hz. A camera recorded the infant’s behavior. The experimenters (P.A. and A.P.) also inspected infants’ behavior and manually marked the approximate awake/sleep state of infants (based on sustained eye opening or movement) during the recording, in order to allow experts (H.N. and P.A.) to score sleep stages after the exams (seeSection 2.4).

### 2.3 Preprocessing

EEG recordings were first bandpass filtered between [0.2–45] Hz, similar to the microstate study in full-term neonates (Khazaei et al., 2021). Note that the high-pass threshold was set slightly higher to better remove low-frequency artifacts. Preprocessing was then performed using an automated pipeline for infants’ continuous EEG (APICE) (Flo et al., 2022). In brief, preprocessing consisted of detecting artifacts on the continuous data where “bad times” and “bad channels” were first defined. Bad times were identified as those with more than 30% of the rejected channels and lasting at least 100 ms. Bad channels were those presenting artifacts during more than 30% of the good times. Artifacts were corrected using target principal component analysis on segments shorter than 100 ms and spatial spherical spline to interpolate bad channels. This resulted in %0.8 ± 0.6 [%0.1 %1.9] corrected data using target principal component analysis and 10 ± 4.4 [5–19] interpolated channels. Finally, artifacts were detected again, and bad times and channels were re-defined. After applying this automated pipeline, recordings were also visually inspected after this step, and obvious remaining artifacts were marked manually. At the end of this step, segments shorter than 8 seconds were rejected from further analyses. All EEG recordings were down-sampled to 250 Hz and re-referenced to the common-average montage before further processing.

### 2.4 Sleep scoring

For assessing the vigilance state, recordings were first transformed into a low-density bipolar montage as in the standard clinical settings. Different vigilance states were then scored by experts in neonatal electrophysiology (H.N. and P.A.). At 0 and 2 mCA, the recordings were categorized into wakefulness, rapid eye movement (REM) sleep (with low voltage irregular or mixed activity patterns), and non-REM (NREM) sleep (*trace alternant*or high voltage slow activity at 0 mCA, and sleep spindles at 2 mCA) (Grigg-Damberger, 2016). Wakefulness was distinguished from REM and NREM sleep, based on manual marks from the recording session and camera videos, and considering sustained eye openings and movements. After sleep scoring and preprocessing, we recovered on average 2.5 ± 1.9 [0.7, 7.9]/6.4 ± 2.5 [1.1, 12.5]/3.4 ± 2.8 [1.0, 10.9] minutes of recordings for wakefulness/REM sleep/NREM sleep at 0 mCA per infant, and similarly 4.1 ± 2.9 [0.8, 11.0]/5.8 ± 2.7 [1.2, 13.7]/4.4 ± 2.7 [0.9, 11.5] minutes of recordings at 2 mCA (as detailed inSection 2.5, not all infants had data in all vigilance states). We only considered comparable vigilance states for age group comparisons. To maintain some homogeneity in terminology, we use REM/NREM labels along the manuscript for both ages, but note that REM/NREM indicate proxies for Active/Quiet sleep at 0 mCA (terms that are more conventionally used at this age). The results for REM/NREM states are presented in the main body of the manuscript and those of wakefulness inSupplementary Information, as they involved shorter recording duration and reduced number of infants.

### 2.5 Microstate analysis

Microstate analysis was performed using the Microstate EEGlab toolbox (Delorme & Makeig, 2004;Poulsen et al., 2018), through the following steps: for each subject, 1,000 random EEG samples (topographical maps) were taken from the global field power maxima separately for each of the vigilance states. These EEG samples were normalized by the average global field power for each individual to account for the interindividual differences in signal strength (due to non-neuronal differences) and were then concatenated across all subjects and partitioned into a fixed number of microstate classes using modified K-means clustering (Pascual-Marqui et al., 1995) without differentiating opposite polarity topographies (polarity invariance). This allowed identifying the microstate classes (i.e., template microstates) at the group-level per each vigilance state. Clustering was restarted 100 times, and each time the modified k-means was iteratively applied until a convergence threshold of 1e-8 (i.e., relative change in error between iterations) was reached.

**Fig. 1.**
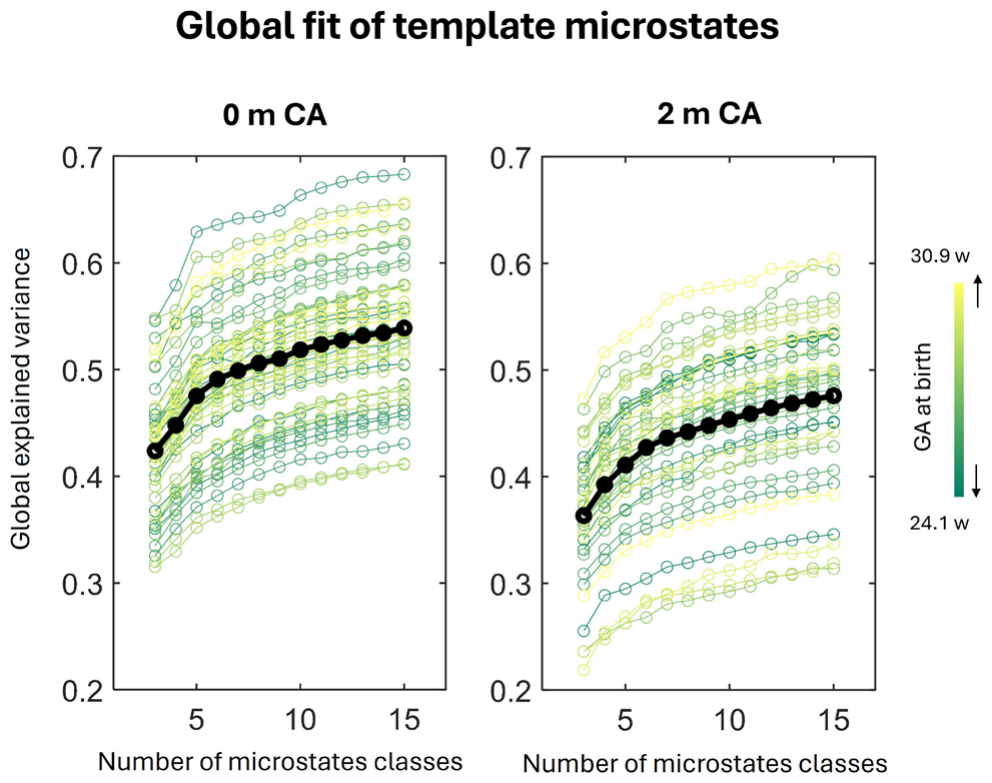
Global fit of the template microstates for different number of microstate classes and different vigilance states. Gain in the global explained variance as a function of the number of microstate classes (3 to 15) for preterm infants at 0 months of corrected age (0 mCA) and 2 mCA in the overall recordings, without separating vigilance states. Each individual infant is color-coded based on the gestational age (GA) at birth. For a number of microstates higher than 7, the gain in the explained variance dropped below 1%.

For the choice of number of microstate classes, we aimed to maintain consistency with the previous work byKhazaei et al. (2021)who reported 7 microstates in full-term neonates. Similarly to their work, we incremented the number of microstate classes between 3 and 15 and estimated how well the template microstates fitted the data by measuring the global explained variance in the data as a function of the number of microstate classes(Fig. 1). The gain in the global explained variance was less than 1% when more than 7 microstate classes were considered, suggesting that considering more classes would not have a major impact on explaining the variance. To obtain comparable measures to this previous study (Khazaei et al., 2021), we thus selected 7 classes of microstates in further analyses. We also verified that the identified microstates did not demonstrate any features typical of heart or muscle artifacts.

The first analysis aimed at comparing microstate characteristics between the preterm and full-term groups in order to identify the potential main effect of prematurity on microstate development. To do so, we focused on identifying the group-level template microstates across both preterm and full-term infants, separately for each vigilance state and each age group (we did not include awake state in this analysis since very few infants in the full-term group had adequate data for this condition). Identifying the template microstates across all infants required accounting for the imbalanced number of subjects in the two groups (more infants in the preterm than the full-term group), in order to avoid biasing the measures to one group. This was achieved by creating five subsets of preterms, where each subset had the same number of infants as the full-term group (0 mCA: n_full-terms_= 9 for REM sleep/n_full-terms_= 10 for NREM sleep; 2 mCA: n_full-terms_= 8 for REM sleep/n_full-terms_= 9 for NREM sleep), including infants from various GA at birth and allowing repetitions across subsets while ensuring that each infant appeared at least in one subset. We identified the 7 template microstates for each of the five preterm subsets completed by the full-term neonates (for each subset: 0 mCA: n_full-terms+preterms_= 18 for REM sleep/n_full-terms+preterms_= 20 for NREM sleep; 2 mCA: n_full-terms+preterms_= 16 for REM sleep/n_full-terms+preterms_= 18 for NREM sleep). The final set of template microstates, for each of the four conditions (age x sleep stage), was obtained by averaging the template microstates across the five corresponding subsets.

In a second step, analyses focused on the whole preterm group alone in order to evaluate the impact of clinical factors related to prematurity as well as maturational changes between 0 and 2 mCA in preterms. The 7 template microstates were identified at the preterm group-level, separately for each vigilance state and each age (0 mCA: n = 17 for awake/n = 41 for REM sleep/n = 21 for NREM sleep; 2 mCA: n = 18 for awake/n = 22 for REM sleep/n = 23 for NREM sleep).

The procedures above identified group-level template microstates for each of the planned analyses, which were then projected back into each individual infant recording while applying a 30 ms temporal smoothing. Back-projecting templates assigned each EEG sample to a microstate class, based on their global topographical dissimilarity invariant to the strength of the signal (Murray et al., 2008). This allowed parsing the continuous EEG recordings of each individual infant into a sequence of microstates, which, in turn, provided statistical metrics describing the microstate dynamics. These metrics included duration (the average life-time of a microstate when activated), occurrence (the frequency of appearance per second), coverage (the fraction of recording time a given microstate is active), and global explained variance (the similarity of EEG samples to a given microstate class). Only results of the analyses for MS duration are presented in the main manuscript, following our hypothesis that higher maturation is reflected by faster brain dynamics and therefore shorter duration. Results for all other metrics are presented inSupplementary Information, as well as analysis of transition probabilities between different microstates, measuring how frequently one microstate is followed by another microstate.

### 2.6 Statistical analyses

#### 2.6.1 Comparing microstates between the preterm and full-term infants: Impact of prematurity

We first evaluated whether the microstates approach is sensitive enough for capturing the effects of prematurity on the brain activity dynamics. To this end, we compared the metrics of microstate dynamics between preterm and full-term infants separately for each age group and sleep state by conducting Analyses of Variance (ANOVA) with each microstate metric (e.g., duration in the main manuscript) as dependent variable, group (preterm/full-term) as between-subject factor, microstate (MS 1 to 7) as within-subject factor, and the two-way interaction group x microstate. This involved conducting 4 ANOVAs per metric (2 age groups, 2 sleep states since wakefulness was not considered).

#### 2.6.2 Comparing microstates between 0 and 2 mCA: Maturation of brain dynamics in preterms

**Fig. 2.**
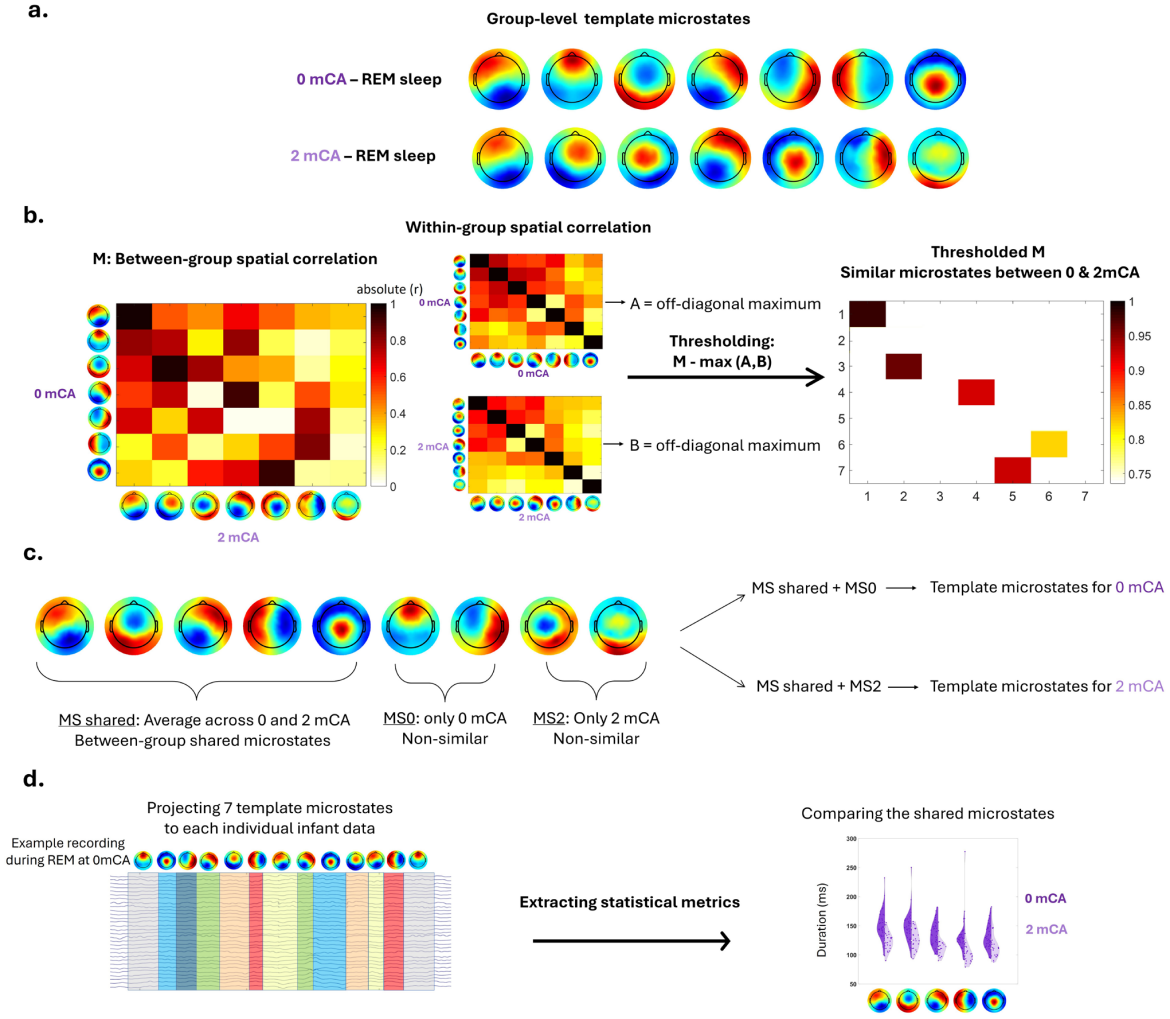
Pipeline comparing microstate characteristics at 0 and 2 mCA in preterms in comparable vigilance states. (a) Group-level template microstates are identified separately for each age group in preterm infants and their head-surface topographical representations are illustrated. (b) Similarity between group-level microstates of the two age groups is computed using spatial correlation irrespective of polarity of the maps. Similarity between group-level microstates is also computed for each group separately (i.e., within-group), and their maximal values are used to threshold the matrix representing the between-group similarity. (c) Similar microstates between the two age groups are averaged, constituting the shared microstates (“MS shared”). Non-similar microstates are kept in each age group (0 mCA/2 mCA → MS0/MS2). The combination of shared and non-similar microstates constituted the final set of 7 template microstates for each age group, among which shared microstates are comparable. (d) The final template microstates for each age group are back projected to each individual infant recording, illustrating activation of different microstates over time. Microstate metrics are extracted for all the microstates and are compared between age groups only for the shared microstates.

We assessed how microstates change with maturation between 0 and 2 mCA, focusing on the preterm group for three reasons: 1) the results from previous analysis suggested group differences, 2) the main scope of this study focused on prematurity, and 3) the reduced number of full-term infants limited analyses on this group alone. The pipeline implemented to compare microstates characteristics at 0 and 2 mCA in preterms is presented inFigure 2and detailed here. We first compared the two ages at the level of template microstates (0 vs. 2 mCA) and by measuring their similarity in terms of spatial distribution (Fig. 2a, b). This was achieved by computing the spatial correlation, that is, absolute Pearson correlation between the vectors of activity values across channels, between the template microstates of the two age groups. This resulted in a correlation matrix, where each value indicated the similarity index between a pair of microstates of the two age groups. To set a threshold for determining similarity of microstates as high or low, we also calculated the spatial correlation between the template microstates within each age group, reasoning that the maximum value of within-group spatial correlations can be considered as the highest spatial similarity between independent states, below which microstates can be considered as independent. Based on this value, we thresholded the two-age-correlation matrix and merged the template microstates with high-similarity by averaging them. This resulted in a set of averaged templates, shared between the two age groups, in addition to non-shared (or non-similar) templates corresponding to the remaining templates with low similarity between the two ages (Fig. 2c). As indicated in the results section, this analysis identified microstates shared between two age groups: 5 for REM sleep, 6 for NREM sleep, and 5 for wakefulness. When projecting back the template microstates to each individual infant, we used the shared and non-shared template microstates for each age group (Fig. 2c, d).

This approach allowed us to compare microstate metrics between the two age groups, focusing on the shared microstates, for each vigilance state. Again, we conducted an ANOVA for each metric (e.g., duration) as dependent variable, age group (0 mCA/2 mCA) as between-subject factor, microstate as within-subject factor, and the two-way interaction age group x microstate. This involved conducting 3 ANOVAs per metric (3 vigilance states).

Note that few infants had longitudinal data for the same vigilance state: only for REM sleep, an adequate number of infants with longitudinal data (n = 21) allowed us to run an additional ANOVA with age group as within-subject factor (i.e., repeated measure) to evaluate whether the results were consistent with the previous analysis with age group as between-subject factor.

As an exploratory analysis, we also investigated whether a structure (i.e., temporal dependency) is observed in the successive activation of microstates, based on transition probabilities (Section 3.2 inSupplementary Information).

#### 2.6.3 Relating microstates at 0 mCA and their 0 → 2 mCA maturation to clinical factors in preterms

To determine how neonatal characteristics at birth and clinical factors during the perinatal period might impact brain maturation, we finally explored the relationships between microstates metrics and the following 5 prematurity risk factors: 1) groups of GA at birth (GA1/GA2/GA3); 2) sex (males/females); 3) binarized risk of small weight for gestational age (yes/no); 4) binarized Kidokoro score (mild abnormality/normal); and 5) binarized neonatal morbidity factor (at least one of the 5 identified non-neurological complications/none). To maximize the statistical power and the number of infants, this analysis focused on the conditions of REM sleep at 0 mCA (n = 41) and at 2 mCA (n = 21-longitudinally).

A first ANOVA (n = 41) assessed the relationship between the MS duration in REM sleep at 0 mCA (focusing on microstates for this vigilance state and age) as dependent variable, the 5 risk factors as independent between-subject factors, and microstate (microstate 1 to 7) as independent within-subject factor, as well as specified two-way interactions (microstate x each of the 5 factors, and GA group x sex; all other two-way interactions were not included because they involved few data points). Note that we also performed a similar ANOVA taking into account, not the binary factor of neonatal morbidity, but the 6-level continuous ratio score (no clinical factor to 5 of them), which required considering the averaged MS duration instead of each MS separately, given the limited sample size.

A second ANOVA (n = 21) evaluated the relationship between the longitudinal difference in microstate duration in REM sleep between 0 and 2 mCA (focusing on shared microstates between the two ages) as dependent variable, risk factors (focusing on GA group, sex, and binarized neonatal morbidity factor) as independent between-subject factors, microstate (microstate 1 to 5) as independent within-subject factor, as well as specific two-way interactions (focusing on microstate x GA group, microstate x sex, and GA group x sex). In this analysis, we did not consider Kidokoro score, the binary factor of small weight for gestational age, as well as other two-ways interactions, because the reduced number of subjects and the reduced inter-individual variability for these factors limited reliable testing.

For all statistical tests, when effects were found to be significant, post hoc tests were carried out using t-tests. And when they involved multiple tests, statistics were corrected for multiple comparisons using the False Discovery Rate (FDR) approach.

## 3 Results

### 3.1 Identifying microstates at different vigilance states

Considering 7 template microstates per age and per sleep states allowed us to describe 0.53/0.59 and 0.45/0.52 of group-level global explained variance for 0 and 2 mCA groups respectively across REM/NREM sleep. We observed a decrease in global explained variance between 0 and 2 mCA (p < 0.005,Fig. 1) and an increase from wakefulness to sleep at both ages (Supplementary Fig. 5). The group-level template microstates for different vigilance states and age groups are presented inSupplementary Figure 8.

As mentioned in the methods section, results for MS metrics other than duration as well as all results for awake state are presented inSupplementary Information(Sections 3 and 5).

### 3.2 Comparing microstates between preterm and full-term infants: Impact of prematurity

Table 2(MS duration) andSupplementary Table 2(other MS metrics) summarize the results of statistical analyses (ANOVA) for the differences between the preterm and full-term groups, at 0 and 2 mCA and for each sleep state.

**Table 2.** Comparison of microstates (MS) duration between Preterm (PT) and Full-Term (FT) infants for different sleep states at 0 and 2 months of Corrected Age (mCA).

**a. REM sleep**			
		**0 mCA**	**2 mCA**
		**Duration**	**Duration**
**Group (PT vs. FT)**		F(1, 48) = 5.7, p = 0.021*	F(1, 28) < 0.1, p = 0.989
**Microstate**		F(6, 288) = 41.2, p < 0.005**	F(6, 168) = 11.1, p < 0.005**
**Group** **x** **Microstate**		F(6, 288) = 2.0, p = 0.070	F(6, 168) = 1.9, p = 0.085
**Post hoc**	MS mean (PT vs. FT):	t = 4.5, p < 0.005**	—

**b. NREM sleep**			
		**0 mCA**	**2 mCA**
		**Duration**	**Duration**
**Group (PT vs. FT)**		F(1, 29) = 1.3, p = 0.263	F(1, 30) < 0.1, p = 0.914
**Microstate**		F(6, 174) = 9.3, p < 0.005**	F(6, 180) = 9.0, p < 0.005**
**Group** **x** **Microstate**		F(6, 174) = 1.1, p = 0.382	F(6, 180) = 3.8, p < 0.005**
**Post hoc**	MS mean (PT vs. FT):	—	—
	MS1 (PT vs. FT):	—	t = 1.2, p = 0.471
	MS2 (PT vs. FT):	—	t = 3.1, p = 0.029*
	MS3 (PT vs. FT):	—	t = −1.5, p = 0.431
	MS4 (PT vs. FT):	—	t = −0.3, p = 0.162
	MS5 (PT vs. FT):	—	t = −0.1, p = 0.798
	MS6 (PT vs. FT):	—	t = 1.3, p = 0.227
	MS7 (PT vs. FT):	—	t = −1.6, p = 0.338

Statistical tests were performed for n = 41/n = 22 preterm versus n = 9/n = 8 full-term infants at 0 mCA/2 mCA respectively during REM sleep, as well as for n = 21/n = 23 preterm versus n = 10/n = 9 full-term infants at 0 mCA/2 mCA respectively during NREM sleep. p Values were corrected for multiple comparisons with FDR approach for each set of post hoc tests, and significant statistical tests are indicated with asterisks (*p < 0.05, **p < 0.005). Comparisons regarding coverage, occurrence, and global explained variance are indicated inSupplementary Table 2.

**Fig. 3.**
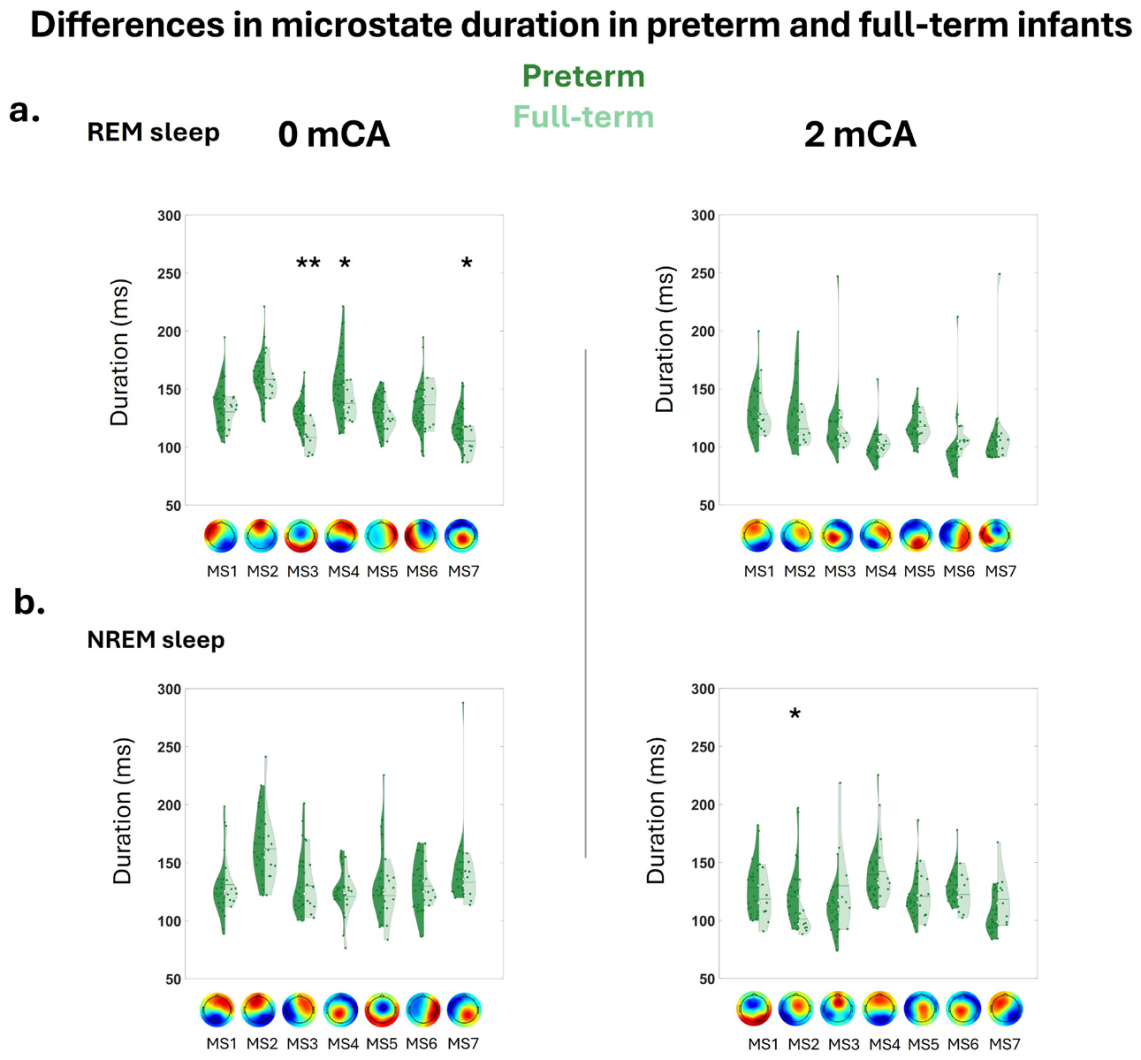
Differences in microstate duration between preterm (dark green) and full-term (light green) infants at 0 mCA (left panel) and 2 mCA (right panel) during different vigilance states: (a) REM sleep; (b) NREM sleep. Asterisks represent significant differences between preterms and full-terms (**p < 0.005, *p < 0.05). For the other microstate metrics (coverage and occurrence), seeSupplementary Figure 1.

At 0 mCA, MS duration in REM sleep showed sensitivity to prematurity: there was a main effect of group (preterm vs. full-term, p = 0.021) and microstate class (p < 0.005) but no interaction between group and microstate class (p > 0.05). Post hoc test showed longer duration, averaged across all MS classes, in preterms than full-terms (p < 0.005) (Fig. 3a). In NREM sleep, we observed a main effect of microstate class (p < 0.005) but no main effect of group or interaction between group and microstate class (all p > 0.05) on MS duration (Fig. 3b).

At 2 mCA, in REM sleep, duration showed a main effect of microstate class (p < 0.005) but no significant main effect of the group or interaction between group and microstate class (all p > 0.05) (Fig. 3a). In NREM sleep, we observed a main effect of microstate class and an interaction between microstate class x group (all p < 0.005), but no main effect of group (p > 0.05) (Fig. 3b). Post hoc tests showed longer microstate duration of MS2 in preterms compared to full-terms (p = 0.029).

### 3.3 Comparing microstates between 0 and 2 mCA: Maturation of brain dynamics in preterms

Table 3(MS duration) andSupplementary Table 3(other MS metrics) summarize the results of statistical analyses for the changes between 0 and 2 mCA, considering different vigilance states and shared MS templates between age groups as detailed in the Methods section andFigure 2.

**Table 3.** Maturation of microstates duration between 0 and 2 mCA for different sleep states.

**a. REM sleep**		
		**Duration**
**Age (0 vs. 2 mCA)**		F(1, 61) = 33.8, p < 0.005**
**Microstate**		F(4, 244) = 14.1, p < 0.005**
**Age** **x** **Microstate**		F(4, 244) = 0.9, p = 0.453
**Post hoc**	MS mean (0 vs. 2 mCA):	t = 5.6, p < 0.005**
	MS1 (0 vs. 2 mCA):	—
	MS2 (0 vs. 2 mCA):	—
	MS3 (0 vs. 2 mCA):	—
	MS4 (0 vs. 2 mCA):	—
	MS5 (0 vs. 2 mCA):	—

**b. REM sleep (longitudinal)**		
		**Duration**
**Age (0 vs. 2 mCA)**		F(1, 20) = 46.1, p < 0.005**
**Microstate**		F(4, 80) = 8.3, p < 0.005**
**Age** **x** **Microstate**		F(4, 100) = 0.6, p = 0.630
**Post hoc**	MS mean (0 vs. 2 mCA):	t = 4.9, p < 0.005**

**c. NREM sleep**		
		**Duration**
**Age (0 vs. 2 mCA)**		F(1, 42) = 6.1, p = 0.018*
**Microstate**		F(5, 210) = 13.1, p < 0.005**
**Age** **x** **Microstate**		F(5, 210) = 5.9, p < 0.005**
**Post hoc**	MS mean (0 vs. 2 mCA):	—
	MS1 (0 vs. 2 mCA):	t = 1.6, p = 0.186
	MS2 (0 vs. 2 mCA):	t = 3.0, p = 0.015*
	MS3 (0 vs. 2 mCA):	t = 0.9, p = 0.389
	MS4 (0 vs. 2 mCA):	t = -2.2, p = 0.066
	MS5 (0 vs. 2 mCA):	t = 4.0, p < 0.005**
	MS6 (0 vs. 2 mCA):	t = 1.4, p = 0.202

Statistical comparisons were performed for n = 41 at 0 mCA versus n = 22 at 2 mCA preterm infants, during REM and for n = 21 at mCA versus n = 23 at 2 mCA preterm infants, during NREM sleep. The statistical tests in (a) and (c) were made cross-sectionally and in (b) for n = 21 infants longitudinally. p Values were corrected for multiple comparisons with FDR approach for each set of post hoc tests, and significant statistical tests are indicated with asterisks (*p < 0.05, **p < 0.005). Comparisons regarding coverage, occurrence, and global explained variance are indicated inSupplementary Table 3.

**Fig. 4.**
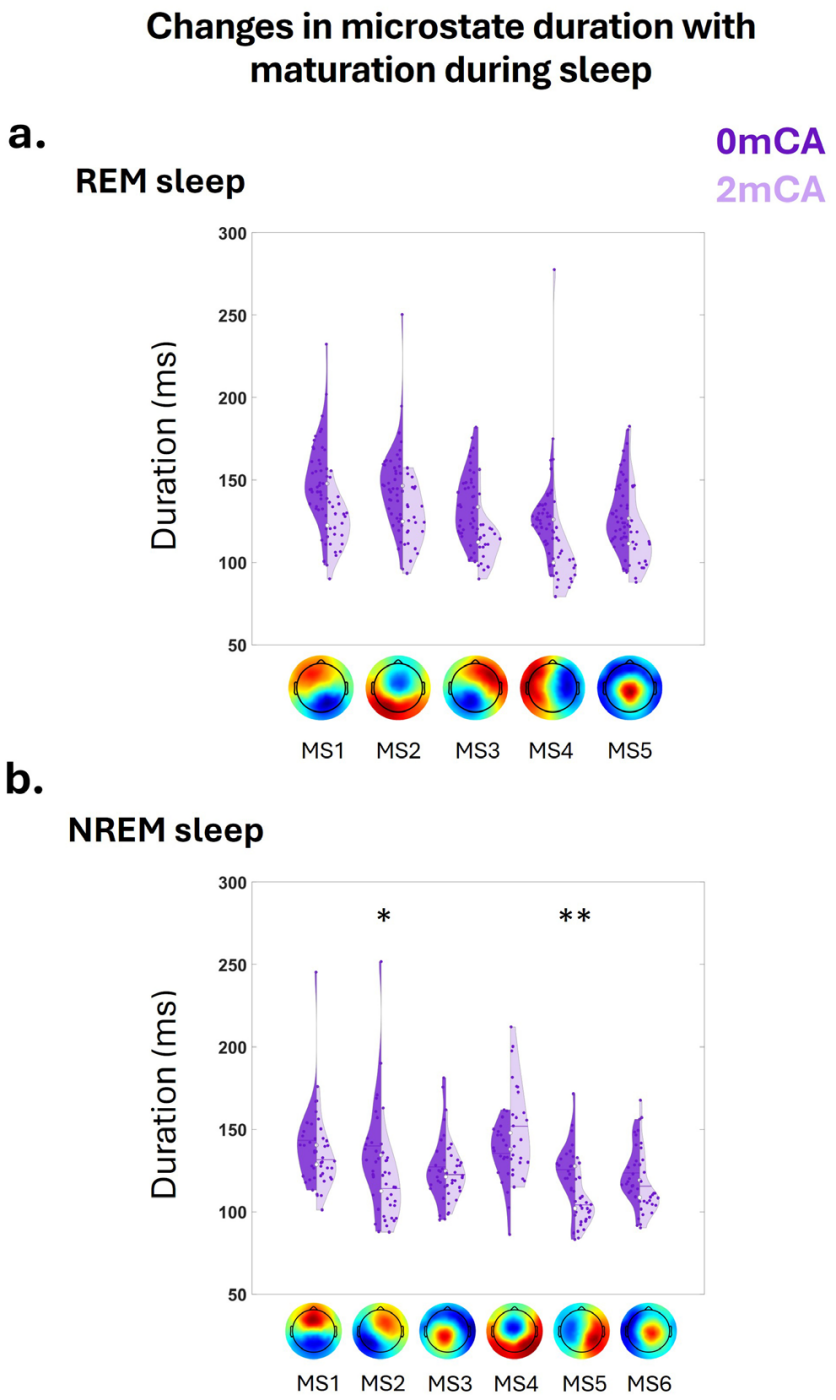
Evolution in microstate duration between 0 mCA (dark purple) and 2 mCA (light purple) in preterms, during different sleep states: (a) for the 5 shared microstates identified in REM sleep; (b) for the 6 shared microstates in NREM sleep. Asterisks represent significant differences between 0 and 2 mCA infants (**p < 0.005, *p < 0.05). For the other microstate metrics (coverage, occurrence, and global explained variance), seeSupplementary Figure 1.

For REM sleep, 5 microstates out of 7 were shared between 0 and 2 mCA and therefore included in the ANOVA (Table 3a). We observed a main effect of age group and microstate class on the MS duration (all p < 0.005) but no interaction between age group and microstate class (p > 0.05). Post hoc tests indicated shorter microstate durations, averaged across all MSc classes, at 2 mCA compared to 0 mCA (p < 0.005,Fig. 4a). When restricting infants to the subgroup with longitudinal data both at 0 and 2 mCA for REM sleep, results were replicated (Table 3b).

For NREM sleep, 6 microstates out of 7 were shared between 0 and 2 mCA and therefore included in the ANOVA (Table 3c). We observed a main effect of age group (p = 0.018) and microstate class (p < 0.005) and an interaction between age group and microstate class (p < 0.005) on MS duration. Post hoc analyses indicated that MS2 and MS5 showed shorter duration at 2 mCA compared to 0 mCA (MS2: p = 0.015, MS5: p < 0.005) (Fig. 4b).

The microstates not shared between the 0 and 2 mCA groups suggested differences in the spatial distribution of network activity. In REM sleep, non-shared microstates showed more abundant posterior and central/posterior topographical distributions at 2 mCA compared to 0 mCA (Fig. 2;Supplementary Fig. 8c) while in NREM sleep, they showed more abundant presence of anterior/posterior topographical distributions at 2 mCA (Supplementary Fig. 8c).

For the sake of simplicity, the transition probabilities between microstates are described in Supplementary Information (Supplementary Fig. 3).

### 3.4 Relating perinatal factors to microstates at 0 mCA and their maturation between 0 and 2 mCA

**Fig. 5.**
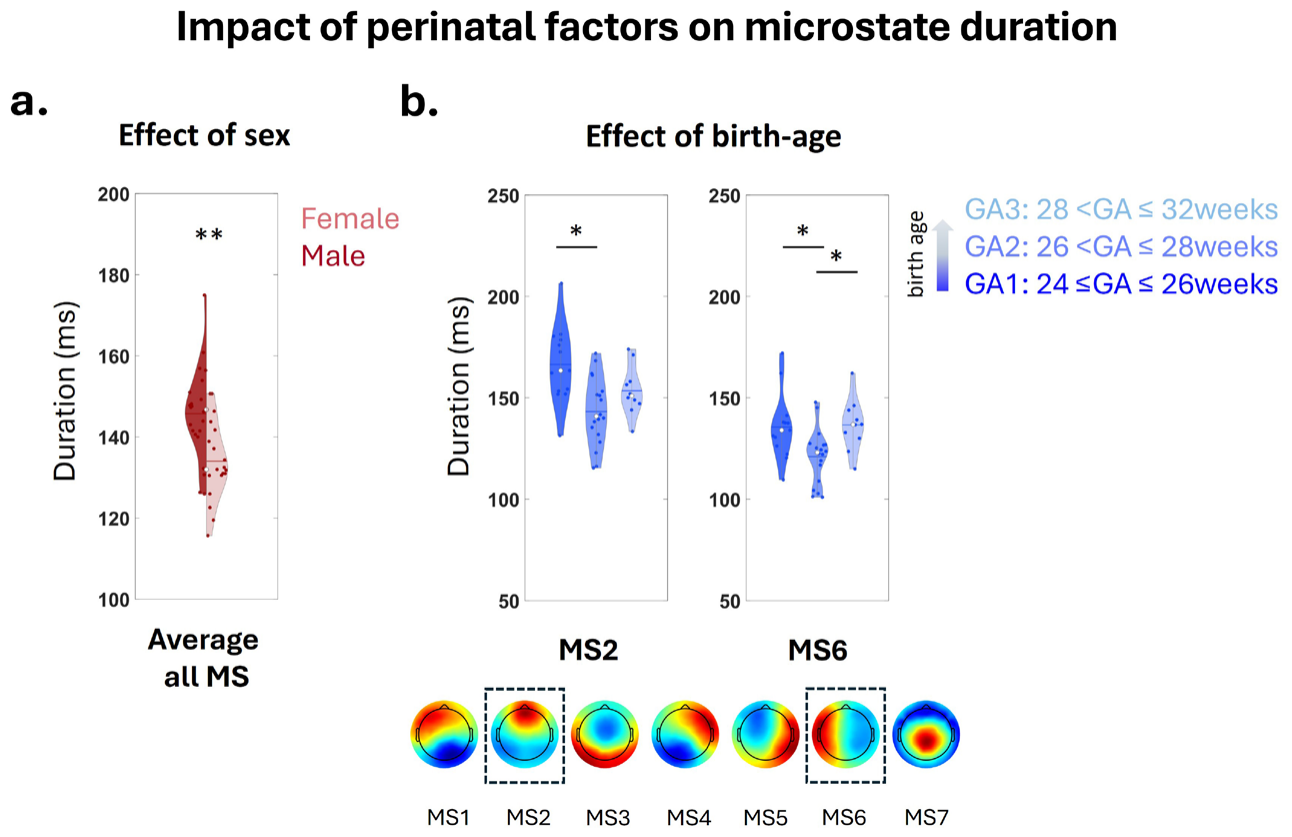
Impact of perinatal factors on microstate duration at 0 mCA for infants born preterm. Impact of (a) Sex, considering the average duration over all MS; (b) Groups of GA at birth for specific MS of REM sleep. Asterisks represent significant differences between Sex and GA groups (**p < 0.005, *p < 0.05).

Finally, we performed statistical analyses to evaluate the impact of perinatal factors on the microstate duration, first at 0 mCA and focusing on REM sleep to allow further longitudinal comparisons at 2 mCA. We observed a main effect of sex (p < 0.005), microstate class (p < 0.005), as well as an interaction between group of GA at birth and microstate class (p = 0.016), but no main effect of group of GA at birth, Kidokoro score, birth weight, neonatal morbidity, or other considered interactions (all p > 0.05) (Fig. 5;Table 4). Post hoc tests revealed longer duration, averaged across all MS classes, in males than females (p < 0.005), longer duration for GA1 compared to GA2 for two MS, but also for GA3 compared to GA2 for one MS (p < 0.05). Complementary analysis considering the averaged MS duration and four groups of GA at birth (GA1, GA2, GA3, full-term) confirmed that preterms had longer duration than full-terms (Supplementary Fig. 4;Supplementary Table 4).

**Table 4:** Impact of perinatal factors on microstates at 0 mCA and their maturation between 0 and 2 mCA.

		**0 mCA**	**0 mCA → 2 mCA**
**GA group**		F(2, 31) = 2.6, p = 0.090	F(2, 14) = 1.9, p = 0.185
**Microstate**		F(6, 203) = 12.6, p < 0.005**	F(4, 68) = 0.7, p = 0.545
**MRI Score**		F(1, 31) = 0.5, p = 0.474	—
**Sex**		F(1, 31) = 15.9, p < 0.005**	F(1, 14) = 2.3, p = 0.151
**Small Weight for GA**		F(1, 31) = 4.0, p = 0.054	—
**Morbidity Risk**		F(1, 31) < 0.1, p = 0.803	F(1, 14) < 0.1, p = 0.871
**GA group** **x** **Sex**		F(2, 31) < 0.1, p = 0.928	F(2, 14) < 0.1, p = 0.956
**Microstate** **x** **GA group**		F(12, 23) = 2.1, p = 0.016*	F(8, 68) = 0.5, p = 0.866
**Microstate** **x** **MRI score**		F(6, 203) = 1.2, p = 0.290	—
**Microstate** **x** **Sex**		F(6, 203) = 1.2, p = 0.321	F(4, 68) = 1.0, p = 0.375
**Microstate** **x** **Small Weight for GA**		F(6, 203) = 0.7, p = 0.619	—
**Microstate** **x** **Morbidity Risk**		F(6, 203) = 0.8, p = 0.536	—
**Post hoc**	MS mean: Female vs. Male	t = -3.5, p < 0.005	—
	MS1: GA1 vs. GA2	t = 0.7, p = 0.734	—
	MS1: GA1 vs. GA3	t = -0.1, p = 0.900	—
	MS1: GA2 vs. GA3	t = -0.9, p = 0.734	—
	MS2: GA1 vs. GA2	t = 3.5, p = 0.005**	—
	MS2: GA1 vs. GA3	t = 2.0, p = 0.074	—
	MS2: GA2 vs. GA3	t = -1.9, p = 0.074	—
	MS3: GA1 vs. GA2	t = -0.9, p = 0.816	—
	MS3: GA1 vs. GA3	t = -0.6, p = 0.816	—
	MS3: GA2 vs. GA3	t = -0.1, p = 0.937	—
	MS4: GA1 vs. GA2	t = 1.6, p = 0.249	—
	MS4: GA1 vs. GA3	t = 1.4, p = 0.249	—
	MS4: GA2 vs. GA3	t < 0.1, p = 0.982	—
	MS5: GA1 vs. GA2	t = 1.5, p = 0.223	—
	MS5: GA1 vs. GA3	t = -0.7, p = 0.488	—
	MS5: GA2 vs. GA3	t = -2.3, p = 0.095	—
	MS6: GA1 vs. GA2	t = 2.6, p = 0.024*	—
	MS6: GA1 vs. GA3	t = -0.2, p = 0.867	—
	MS6: GA2 vs. GA3	t = -3.0, p = 0.021*	—
	MS7: GA1 vs. GA2	t = -0.7, p = 0.751	—
	MS7: GA1 vs. GA3	t = 0.3, p = 0.751	—
	MS7: GA2 vs. GA3	t = 0.9, p = 0.751	—

The relationships between microstate duration at 0 mCA (n = 41, left column) or the longitudinal changes in microstate duration between 0 mCA and 2 mCA (n = 21, right column) and different prematurity risk factors were evaluated for REM sleep activity. These factors included group of GA at birth (GA1/GA2/GA3); sex; binarized risk of birth weight indicating small for GA; MRI Kidokoro score; and binarized neonatal morbidity factor summarizing non-neurological complications. At 2 mCA, only main effects and interactions with adequate data points were considered. p Values were corrected for multiple comparisons with FDR approach for each set of post hoc tests, and significant statistical tests are indicated with asterisks (*p < 0.05, **p < 0.005).

For longitudinal changes in MS duration from 0 to 2 mCA, during REM sleep, we did not observe any significant effect (Table 4;Supplementary Table 4).

## 4 Discussion

In this study, we used an original microstate approach to characterize the fast transient dynamics of EEG activity in preterm and full-term infants while considering different vigilance states. We reported three sets of results showing that this approach is sensitive for capturing functional aspects of brain maturation and dysmaturation. First, by comparing preterms and full-terms, we identified alterations at term-equivalent age that also persisted at 2 mCA, indicating the impact of prematurity on early brain activity patterns. Second, comparing EEG dynamics between 0 and 2 mCA in preterms with a robust approach allowed us to identify the presence of both similar (i.e., shared between age groups) and unsimilar microstates in all vigilance states. For microstates similar between age groups, we observed a shortening of microstate duration, as well as changes in the coverage, occurrence, global explained variance, and the transition patterns of microstates (Supplementary Information). These highlighted an evolution in the spatio-temporal properties of brain activity with maturation. Third, we showed that the inter-individual differences in the temporal dynamics of microstates in preterms are impacted by gestational age at birth and sex, with longer microstate duration in infants with lower birth age and in males. This confirmed the sensitivity of the implemented approach in capturing the effects of risk factors on early brain activity.

### 4.1 Brain dynamics are impacted by prematurity and dysmaturation

Comparing preterm and full-term infants indicated that the microstate dynamics, as captured with MS duration and other metrics, are impacted by prematurity, particularly at TEA. These alterations were primarily observed in terms of slower dynamics in preterms (i.e., longer duration and lower occurrence rate of microstates), which may find their roots in less mature brain structures. Such slower dynamics might reflect less advanced or altered myelination of white matter pathways (Neumane et al, 2022), as this mechanism accelerates the conduction of neural impulses and impacts the speed of brain responses during development (Dubois et al., 2008), and/or to less complex cortical microstructure with reduced dendritic arborization and synaptogenesis (Dimitrova et al., 2021;Gondova et al., 2023), as this might negatively impact neural activity synchronization (Dupont et al., 2006). These cerebral alterations in preterms at TEA may, therefore, affect the network-wide activity patterns we measured with the MS approach. Similar slower brain dynamics has also been recently reported in infants with hypoxic-ischemic encephalopathy (Khazaei et al., 2023), confirming that perinatal insults delay the development of network-wide brain activity. However, direct links between the structural and functional alterations remain to be established, potentially by delineating different brain networks that mature progressively during infancy (Dubois et al., 2014,^2016^). In line with this proposition, we also observed that the impact of prematurity differed across microstate classes (interaction group x microstate class) in some conditions (i.e., NREM at 2 mCA), which suggests that not all functional networks are impacted in a similar way. This is further in agreement with the well-known asynchronous maturation pattern of the different brain networks (Dubois et al., 2014;Yakovlev & Lecours, 1967), suggesting that distinct time windows of vulnerability (Wu et al., 2017) lead to differential adverse impacts across cerebral networks (Neumane et al., 2022). Altogether, these findings encourage studying structure-function relations and alterations by considering different networks separately.

Beyond prematurity, we also aimed to evaluate to which extent specific perinatal and clinical factors might impact the development of brain activity dynamics in infants. Previous studies have identified several risk factors for poorer outcomes related to prematurity (Allen, 2008;O’Driscoll et al., 2018;Pierrat et al., 2021). Nevertheless, our sample size limited the possibility to test multiple associations, so we focused on the factors that supposedly have a major impact. First, GA at birth appeared to modulate the microstate dynamics and maturation: preterm infants within GA1 group (24 w ≤ GA ≤ 26 w) showed slower dynamics than GA2 group (26 w < GA ≤ 28 w) and full-term infants (Section 4.1 inSupplementary Information), in a comparable way as the effect of preterm versus full-term birth. Yet, while our preterm cohort had quite uniform sampling of gestational ages at birth between 24 and 32 weeks, we did not observe a dose-dependent effect of gestational age on microstate duration, as GA3 group (28 w < GA ≤ 32 w) did not show shorter MS duration compared to GA1 and GA2 groups. This might be explained by the heterogeneity of the cohort clinical characteristics across GA groups. In fact, only infants due to have a clinical MRI at TEA were included in our study, either because they were born before 28 w GA (GA1 and GA2 groups) and/or because of clinical/neurological suspicions (GA3 group). Thus, contrarily to GA1 and GA2 groups, GA3 group was not an exhaustive representation of preterm infants born between 28 and 32 w GA, even though no infant showed moderate or severe brain abnormalities according to MRI Kidokoro score. Importantly, GA at birth had a disparate impact across the different microstates, suggesting that different networks have different time windows of vulnerability over the gestational period (Wu et al., 2017). In future studies, it would be interesting to characterize such an effect by considering GA as a continuous variable in a larger group of infants, including some with moderate prematurity (GA at birth between 32 and 37 w).

We also evaluated the impact of sex on microstates dynamics, and our results of slower dynamics in males seemed consistent with the large clinical and epidemiological evidence of higher vulnerability of males compared to females over this specific period of development (Hintz et al., 2006;Kelly et al., 2023b;Perivier et al., 2016). These results further suggested the sensitivity of the microstate approach to highlight some inter-individual variability across infants, that might be interesting to target for individualized interventions. Nevertheless, controlling for the known sex differences in brain size might be an important aspect in future studies to disentangle the effects of sex and brain anatomical characteristics on brain dynamics.

Other clinical risk factors of adverse outcomes related to prematurity were also considered (small birth weight, respiratory, intestinal or infectious complications, and brain abnormalities) but none were shown to impact the brain dynamics in our study. Nevertheless, this may be related to the lack of large variability in our cohort of infants and to the limited sample size which constrained us to binarize factors and combine some into a composite morbidity score. It is also possible that some of the assessed clinical factors were not reflective of maturational changes captured with EEG dynamics. In particular, the MRI Kidokoro score is supposed to capture macrostructural abnormalities related to global and regional volumetric growth as well as brain lesions but may not be sensitive to diffuse microstructural alterations impacting the network functional activity.

### 4.2 Maturation changes the spatio-temporal brain dynamics in preterm infants

Comparing the dynamics of the brain activity between 0 m and 2 mCA indicated an evolution in terms of distinctive spatial topographies of activity among the dominant microstates, as well as major impact on temporal properties of microstates already developed early on. The presence of a few non-similar MS across groups might further suggest a reorganization of functional network activities during this period of intense maturation in cortical regions and white matter pathways promoting functional specialization of networks (Dubois et al., 2014;Gao et al., 2015;Keunen, Counsell, Benders, 2017). For example, the appearance of a majorly posteriorly-distributed MS at 2 mCA in REM sleep might relate to the intense post-natal development of the visual system at these ages (Dubois et al., 2008,^2014^).

The observed major shortening of microstate duration between 0 and 2 mCA is also in agreement with a recent study of preterm infants indicating acceleration of MS dynamics between the preterm and term period [250–300 ms at 30 w PMA to 150–200 ms after 37 w PMA] (Hermans et al., 2024). Interestingly, the average duration of the microstates in our study, 140 ms at 0 mCA and 112 ms at 2 mCA, is in the intermediate range between the preterm period (Hermans et al., 2024) and childhood with microstate durations reported between 60 to 90 ms (Bagdasarov et al., 2022;Koenig et al., 2002;Takarae et al., 2022). This suggests a continuous change in the duration of MS from birth through childhood. The shortening of MS duration, together with the increase in the occurrence rate of MS, indicates an acceleration of the brain dynamics which could reflect the intensive myelination of white matter pathways (Dubois et al., 2008), and/or maturation of cortical microstructure (Dimitrova et al., 2021;Gondova et al., 2023) around TEA and first post-term weeks, resulting in an acceleration and better synchronization of neural dynamics. Several developmental changes concerned microstates with an anterior/posterior distribution of the brain activity, a spatial organization also reported to show alterations of functional connectivity patterns in preterms (Omidvarnia et al., 2014;Tokariev et al., 2019). An interesting direction for future investigations would be to address how the functional connectivity patterns identified with EEG relate to the dynamics of microstates and their maturation in early development (seeKhazaei et al., 2023).

While maturational changes between 0 and 2 mCA at REM affected the duration of all MS classes, at NREM sleep these changes concerned some MS classes which might, in part, reflect the maturation changes in sleep structure and in particular the appearance of sleep spindles in NREM sleep after 43 weeks of post-menstrual age (Grigg-Damberger, 2016).

Besides metrics describing individual microstates, we also explored the transitions between microstates (Section 3.2 inSupplementary Information) which offer insights into the structure of the temporal dependencies in brain dynamics. At both ages, the transitions between the microstates had a non-random structure, with most microstates tending to transition into their “favorite” microstates (one or more than one). The pattern of such transitions evolved with development between 0 and 2 mCA, which could suggest a reorganization of the brain functional dynamics, showing an evolution in the continuous shifting between transient network activities. Beyond microstates, dynamic aspects of brain activity have also been described for functional brain networks identified with fMRI at the temporal resolution of the BOLD effect (França et al., 2024), and with functional ultrasound imaging (Baranger et al., 2021), indicating their alterations in preterms before and at TEA compared to full-terms. Here, we could not compare the transition patterns of microstates between the preterm and full-term groups, because of the limited sample size of the latter group.

### 4.3 Methodological considerations for the characterization of microstate dynamics in infants

First, across all analyses, we ensured that the group-level microstate templates were not biased toward a subgroup of infants. For the preterms versus full-terms comparison, we presumed the microstate templates should be similar between the preterm infants at TEA and full-term neonates, given that the group-level microstates identified in the whole preterm group during sleep were qualitatively similar to those of a previous study in full-term asleep neonates (Khazaei et al., 2021). Yet, to ensure that the initially-defined templates were equally representative of both groups and unbiased toward certain GA at birth, we implemented a strategy iterating over subgroups of preterms with the same number as full-terms and with various GA. Consistently, no correlation between GA and the metric of global explain variance was then found. Similarly, we ensured that comparing microstate metrics between 0 and 2 mCA infants relied on an unbiased set of microstates. Since no previous work studied microstates in 2-month-old infants, we implemented a data-driven approach to measure the templates similarity across groups and we considered shared (i.e., similar) microstates for further analyses.

Moreover, we took into account that brain activity might differ across vigilance states (Dereymaeker et al., 2017), given their crucial role in modulating developmental and learning mechanisms early in life (Li et al., 2017). We performed templates identification and analyses for each state separately. We then observed an increase in global explained variance from awake to REM sleep and further into NREM sleep, which is consistent with previous reports in full-term neonates in active versus quiet sleep (Khazaei et al., 2021) and suggests a global reduction in complexity of brain dynamics with progression in sleep depth. While such effects might vary across microstates, as shown in neonates (Khazaei et al., 2021, see alsoKhazaei et al., 2023) and adults (Brodbeck et al., 2012), evaluating the precise impact of vigilance states was beyond the scope of our study.

Besides, our study benefited from HD-EEG recordings that are suggested to improve reliability of microstates analysis (Zhang et al., 2021). Interestingly, the microstates we identified in preterms at TEA were similar to those of full-term asleep neonates reported in the previous study in similar sleep stages (Khazaei et al., 2021), suggesting that such an approach is quite reproducible across different study settings, neonatal populations, and recording systems, and only with a few minutes of recording (Bagdasarov et al., 2024). This opens up the perspective of implementing microstate templates by age group, available to the scientific and clinical community for future multi-site studies for example.

Finally, our study was exploratory in taking a step toward characterizing early maturation and dysmaturation of microstate dynamics reflecting the brain activity. Since our study was limited by the sample size of preterms (n = 43 but with inter-individual variability in clinical characteristics) and full-terms (n = 14) and uncomplete longitudinal evaluations, future studies in larger cohorts could better address relationships with neonatal factors. A crucial next step is to tackle the links between microstates and the underlying cerebral networks for understanding the specific functional relevance of different microstates in the developing infant brain.

## 5 Conclusions

This study implemented a dedicated strategy for the quantitative evaluation of brain spontaneous activity in preterm infants using the EEG microstate approach, highlighting fundamental features that describe the maturation of the brain dynamics and show sensitivity to perinatal factors. Such an approach might offer novel potential diagnostic markers for understanding the heterogeneity of atypicalities and developmental trajectories over the early post-natal period of intense neuroplasticity, when interventions building on compensatory mechanisms could improve the neurodevelopmental outcomes (Livingston & Happe, 2017). Although beyond the scope of current study, this encourages future research to perform similar analysis in larger groups of full-term neonates in order to characterize the normative developmental trajectories required for a better identification of atypicalities in preterm infants with a wide inter-individual variability.

## Data and Code Availability

Preprocessed data and the custom Matlab code implementing the pipelines will be available upon reasonable request to the corresponding author and within the limits of ethical agreement on the sharing of individual data.

## Author Contributions

Conceptualization: P.A., V.B., and J.D.; Data curation: P.A., A.P., L.D., N.E., C.G., and J.D.; Formal analysis: P.A., H.N.; Funding acquisition: R.D., M.B.-R., V.B., and J.D.; Investigation: P.A., M.A., R.D., M.B.-R., V.B., and J.D.; Methodology: P.A., H.N., S.N., C.K., A.K., V.B., and J.D.; Project administration: E.H., L.H.-P., A.H., M.A., M.B.-R., V.B., and J.D.; Resources: A.L., O.S., M.A., C.D., R.D., M.B.-R., V.B., and J.D.; Supervision: J.D. and V.B.; Validation: P.A., C.K., A.K., and J.D.; Visualization: P.A.; Writing—original draft: P.A. and J.D.; and Writing—review & editing: All.

## Declaration of Competing Interest

The authors declare that they have no financial conflict of interest with the content of this article.

## Supplementary Material

Supplementary Material
