# Supplementary material for: Characterizing the temporal dynamics and maturation of brain activity during sleep: An EEG microstate study in preterm and full-term infants
Source: Imaging Neurosci (Camb). 2025 Jan 29;3:imag_a_00450. doi: 10.1162/imag_a_00450 (PMC12319786; doi:10.1162/imag_a_00450)
Supplement: Supplementary Material [file imag_a_00450-supp.pdf]

Supplementary Information

Characterizing the temporal dynamics and maturation of brain activity during sleep: an EEG microstate study in preterm and full-term infants.

Parvaneh Adibpour[1,2 *], Hala Nasser[1,3], Amandine Pedoux[4], Laurie Devisscher[1,2], Nicolas Elbaz[5], Chloé Ghozland[6], Elodie Hinnekens[7], Sara Neumane[1,2,8], Claire Kabdebon[9], Aline Lefebvre[4], Anna Kaminska[1,10], Lucie Hertz-Pannier[1,2], Alice Heneau[6], Olivier Sibony[11], Marianne Alison[1,5], Catherine Delanoë[3], Richard Delorme[4], Marianne Barbu-Roth[7], Valérie Biran[1,6], Jessica Dubois[1,2]

1. Université Paris Cité, INSERM, NeuroDiderot, F-75019 Paris, France
2. Université Paris Saclay, CEA, NeuroSpin, UNIACT, F-91191 Gif-sur-Yvette, France
3. Assistance Publique-Hôpitaux de Paris - APHP, Robert-Debré University Hospital, Department of Physiology – Functional Explorations, F-75019 Paris, France
4. APHP, Robert-Debré University Hospital, Department of Child and Adolescent Psychiatry, F-75019 Paris, France
5. APHP, Robert-Debré University Hospital, Department of Pediatric Radiology, F-75019 Paris, France
6. APHP, Robert-Debré University Hospital, Neonatal Intensive Care Unit, F-75019 Paris, France
7. Université Paris Cité, CNRS, Integrative Neuroscience and Cognition Center, F-75005 Paris, France
8. Université Paris Saclay - Université Versailles St Quentin, APHP, Raymond Poincaré University Hospital, Pediatric Physical Medicine and Rehabilitation Department, Garches, France
9. Université Aix-Marseille, CNRS, Institute of Language, Communication and the Brain, Marseille, France
10. APHP, Necker-Enfants Malades University Hospital, Department of Clinical Neurophysiology, F-75015 Paris, France
11. APHP, Robert-Debré University Hospital, Department of Gynecology-Obstetrics, F-75019 Paris, France

* Corresponding author:

Parvaneh Adibpour

NeuroDiderot Unit UMR1141
Inserm, Université Paris Cité
CEA/SAC/NeuroSpin/UNIACT
Bat 145, point courrier 156
Gif-sur-Yvette, 91191, FRANCE

Email : parvaneh.adibpour@inserm.fr

Supplementary Information

1. Participants information

SI Table 1. Detailed neonatal characteristics and clinical risk factors for each of the preterm infants. These include groups of GA at birth (GA1/GA2/GA3), sex (males/females), binarized risk of birth weight indicating small for gestational age (yes/no), binarized MRI Kidokoro score (mild/normal), as well as information regarding five categories of non-neurological complications at NICU. These complications were considered for chronic lung disease (yes/no), use of invasive mechanical ventilation for more than 1 day (yes/no), necrotizing enterocolitis (yes/no), parenteral nutrition for more than 3 weeks (yes/no) and sepsis (yes/no).

Subjects	GA at birth	GA category	Sex	Small weight for GA	MRI Kidokoro score	Chronic lung disease	Mechanical ventilation>1day	Necrotizing enterocolitis	Parenteral nutrition>3weeks	Sepsis
1	26.6	2	M	No	Normal	No	No	No	No	Yes
2	27.0	2	F	No	Normal	No	Yes	Yes	No	No
3	27.3	2	F	No	Normal	No	No	No	No	Yes
4	27.9	2	M	No	Mild	No	No	No	No	No
5	25.7	1	M	No	Normal	No	No	No	No	Yes
6	25.7	1	M	No	Normal	No	No	No	No	Yes
7	25.1	1	F	Yes	Normal	Yes	Yes	No	Yes	Yes
8	26.4	2	M	No	Normal	No	No	No	Yes	Yes
9	26.4	2	F	No	Mild	Yes	No	No	Yes	Yes
10	26.6	2	M	No	Normal	No	No	No	No	Yes
11	26.6	2	M	No	Normal	No	No	No	No	Yes
12	29.9	3	M	Yes	Normal	No	Yes	Yes	Yes	Yes
13	29.7	3	F	No	Normal	No	No	No	No	Yes
14	25.9	1	F	No	Normal	No	No	No	No	Yes
15	25.9	1	M	No	Normal	Yes	No	No	Yes	Yes
16	25.9	1	F	No	Mild	No	No	No	No	Yes
17	27.4	2	M	No	Mild	Yes	No	No	No	Yes
18	30.9	3	M	No	Normal	No	No	No	No	No
19	26.0	1	M	No	Normal	Yes	No	No	Yes	Yes
20	25.1	1	F	No	Normal	Yes	Yes	No	No	Yes
21	27.9	2	M	No	Normal	Yes	No	No	No	No
22	28.1	3	F	Yes	Normal	Yes	Yes	Yes	Yes	Yes
23	27.7	2	M	No	Normal	No	No	No	No	No
24	28.9	3	F	No	Normal	Yes	Yes	No	Yes	Yes
25	29.7	3	M	No	Normal	Yes	Yes	No	Yes	Yes
26	24.3	1	F	No	Normal	Yes	Yes	No	Yes	Yes
27	26.7	2	F	No	Normal	Yes	No	No	No	No
28	25.0	1	F	No	Normal	Yes	Yes	Yes	Yes	Yes
29	27.1	2	M	No	Normal	Yes	Yes	No	Yes	No
30	28.6	3	M	Yes	Normal	Yes	No	No	No	Yes
31	29.4	2	F	No	Normal	Yes	No	No	Yes	Yes
32	27.6	2	M	Yes	Normal	Yes	No	No	No	Yes
33	26.3	2	F	No	Normal	Yes	Yes	No	Yes	Yes
34	26.7	2	M	No	Normal	Yes	No	No	No	Yes
35	28.3	3	M	No	Normal	Yes	Yes	No	Yes	Yes
36	24.6	1	M	Yes	Normal	Yes	Yes	No	Yes	Yes
37	24.1	1	M	No	Mild	Yes	Yes	Yes	Yes	Yes
38	30.6	3	M	Yes	Mild	No	No	No	No	No
39	26.3	2	F	No	Normal	Yes	Yes	Yes	Yes	Yes
40	27.1	2	F	No	Normal	Yes	Yes	No	Yes	No
41	28.1	3	F	No	Mild	No	No	No	No	No
42	25.1	1	F	No	Normal	Yes	Yes	Yes	Yes	Yes
43	30.9	3	F	Yes	Normal	No	No	No	No	No

2. Comparing microstates between preterm and full-term infants: Impact of prematurity on MS coverage and occurrence and global explained variance

SI Table 2. Comparison of microstates metrics (coverage, occurrence and global explained variance-GEV) between Preterm (PT) and Full-Term (FT) infants for different sleep states. Statistical tests were performed for n=41/n=22 preterm vs n=9/n=8 full-term at 0mCA/2mCA in REM sleep as well as for n=21/n=23 preterm vs n=10/n=9 full-term infants at 0mCA/2mCA in NREM sleep. P values were corrected for multiple comparisons with FDR approach for each set of post hoc tests, and significant statistical tests are indicated with asterisks (* p<0.05, ** p<0.005).

a. REM sleep

		0mCA			2mCA		
		**Coverage**	**Occurrence**	**GEV**	**Coverage**	**Occurrence**	**GEV**
**Group (PT vs FT)**		F(1,48) = 4.0, p = 0.052	F(1,48) = 2.7, p = 0.116	F(1,48) = 0.2, p = 0.667	F(1,28) = 0.7, p = 0.405	F(1,28) < 0.1, p = 0.837	F(1,28) = 0.2, p = 0.689
**Microstate**		F(6,288) = 35.2, p < 0.005**	F(6,288) = 28.3, p < 0.005**	F(6,288) =52.2, p < 0.005**	F(6,168) = 25.0, p < 0.005**	F(6,168) = 45.4, p < 0.005**	F(6,168) = 15.6, p < 0.005**
**Group x Microstate**		F(6,288) = 2.9, p = 0.009*	F(6,288) = 4.3, p < 0.005**	F(6,288) = 2.0, p = 0.062	F(6,168) = 2.6, p = 0.018*	F(6,168) = 3.9, p < 0.005 **	F(6,168) = 1.8, p = 0.094
**Post hoc**	MS mean(PT vs FT):						
	MS1(PT vs FT):	t = -0.2, p = 0.824	t = -0.8, p = 0.424		t =- 0.2, p = 0.897	t = -0.5, p = 0.750	
	MS2(PT vs FT):	t = -1.1, p = 0.485	t = -2.1, p = 0.098		t = 3.0, p = 0.057	t = 2.4, p = 0.079	
	MS3(PT vs FT):	t = 3.5, p = 0.020*	t = 2.5, p = 0.089		t = 2.2, p = 0.119	t = 2.6, p = 0.079	
	MS4(PT vs FT):	t = 0.5, p = 0.751	t = -1.4, p = 0.227		t = -0.8, p = 0.624	t = -0.6, p = 0.720	
	MS5(PT vs FT):	t = -0.7, p = 0.668	t = -2.2, p = 0.098		t = -0.1, p = 0.897	t < -0.1, p = 0.988	
	MS6(PT vs FT):	t = -2.0, p = 0.176	t = -2.9, p = 0.089		t = -1.9, p = 0.164	t = -2.5, p = 0.079	
	MS7(PT vs FT):	t = 2.2, p = 0.164	t = 1.5, p = 0.211		t = -1.3, p = 0.397	t = -1.5, p = 0.259	

b. NREM sleep

		0mCA			2mCA		
		**Coverage**	**Occurrence**	**GEV**	**Coverage**	**Occurrence**	**GEV**
**Group (PT vs FT)**		F(1,29) = 3.9, p = 0.058	F(1,29) = 0.1, p = 0.693	F(1,29) = 0.6, p = 0.445	F(1,30) = 0.7, p = 0.396	F(1,30) < 0.1, p = 0.987	F(1,30) = 0.9, p = 0.6
**Microstate**		F(6,174) = 13.2, p < 0.005**	F(6,174) = 12.3, p < 0.005**	F(6,174)= 23.4, p < 0.005**	F(6,288) = 13.5, p < 0.005**	F(6,180) = 19.3, p < 0.005**	F(6,180) = 18.9, p < 0.005**
**Group x Microstate**		F(6,174) = 1.5, p = 0.174	F(6,174) = 1.7, p = 0.125	F(6,174) = 1.3, p = 0.254	F(6,180) = 4.3, p < 0.005**	F(6,180) = 4.4, p < 0.005**	F(6,180) = 3.2, p < 0.005**
**Post hoc**	MS mean(PT vs FT):						
	MS1(PT vs FT):				t = 0.9, p = 0.457	t = 0.6, p = 0.527	t = 0.2, p = 0.863
	MS2(PT vs FT):				t = 4.5, p < 0.005**	t = 3.3, p = 0.019*	t = 4.2, p < 0.005**
	MS3(PT vs FT):				t = -2.2, p = 0.139	t = -3.1, p = 0.021*	t = -1.9, p = 0.222
	MS4(PT vs FT):				t = -0.9, p = 0.457	t = -1.1, p = 0.440	t = -0.8, p = 0.614
	MS5(PT vs FT):				t = 0.6, p = 0.553	t = 1.0, p = 0.440	t = 0.6, p = 0.673
	MS6(PT vs FT):				t = 1.2, p = 0.457	t = 0.9, p = 0.442	t = 0.9, p = 0.614
	MS7(PT vs FT):				t = -2.1, p = 0.139	t = -1.9, p = 0.177	t = -2.0, p = 0.222

Differences in microstate metrics in preterm and full-term infants

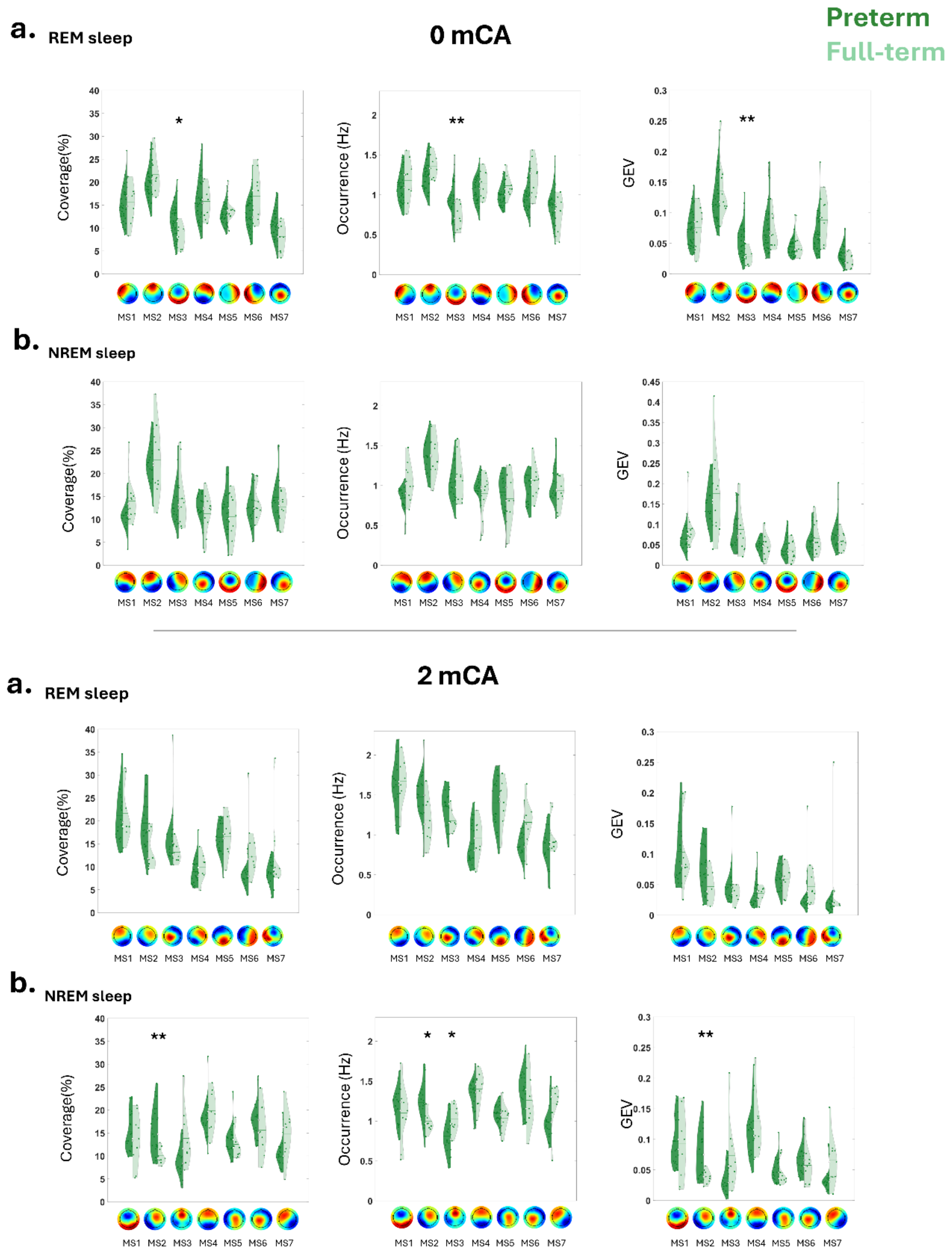

SI Figure 1. Changes in microstate metrics (coverage, occurrence and global explained variance-GEV) between preterms (dark green) and full-terms (light green) in **a.** REM sleep; **b.** NREM sleep. Asterisks represent significant differences between preterm and full-term infants (** p<0.005, *p<0.05).

3. Comparing microstates between 0mCA and 2mCA: Impact of maturation on MS dynamics in preterms

3.1. Maturation of individual microstate metrics

SI Table 3. Maturation of microstate metrics (coverage, occurrence and global explained variance-GEV) between 0mCA and 2mCA during sleep states. Statistical comparisons were performed for n=41 at 0mCA vs n=22 at 2mCA preterm infants during REM and for n=21 at 0mCA vs n=23 at 2mCA preterm infants during NREM sleep. The statistical tests in a. and c. were made cross-sectionally and in b. for n=21 infants longitudinally. P values were corrected for multiple comparisons with FDR approach for each set of post hoc tests, and significant statistical tests are indicated with asterisk (* p<0.05, ** p<0.005).

a. REM sleep

		Coverage	Occurrence	GEV
**Age (0 vs 2mCA)**		F(1,61) = 18.1, p < 0.005**	F(1,61) = 58.4 p < 0.005**	F(1,61) = 0.1, p = 0.902
**Microstate**		F(4,244) = 16.1, p < 0.005**	F(4,244) = 17.4, p < 0.005**	F(4,244) = 26.3, p <0.005**
**Age x Microstate**		F(4,244) = 1.0, p = 0.401	F(4,244) = 5.9, p < 0.005**	F(4,244) = 2.7, p = 0.029*
**Post hoc**	MS mean (0 vs 2mCA):	t =- 3.6 , p<0.005		
	MS1(0 vs 2mCA):		t = - 4.8, p < 0.005**	t = 1.4, p = 0.307
	MS2(0 vs 2mCA):		t = -5.0, p < 0.005**	t = -2.2, p = 0.165
	MS3(0 vs 2mCA):		t = -4.6, p < 0.005**	t = 1.0, p = 0.417
	MS4(0 vs 2mCA):		t = -1.3, p = 0.207	t = 0.7, p = 0.460
	MS5(0 vs 2mCA):		t = -4.6, p < 0.005**	t = -1.3, p = 0.307

b. REM sleep (longitudinal)

		Coverage	Occurrence	GEV
**Age (0 vs 2mCA)**		F(1,20) = 4.2, p = 0.044*	F(1,20) = 80.9, p < 0.005**	F(1,20) < 0.1, p = 0.803
**Microstate**		F(4,80) = 11.6, p <0.005**	F(4,80) = 15.2, p < 0.005**	F(4,80) = 17.5, p <0.005**
**Age x Microstate**		F(4,100) = 1.0, p = 0.405	F(4,100) = 3.6, p = 0.009*	F(4,100) = 2.6, p = 0.040*
**Post hoc**	MS mean (0 vs 2mCA):	t =- 3.3 , p<0.005		
	MS1(0 vs 2mCA):		t = -4.3, p < 0.005**	t = 1.7, p = 0.235
	MS2(0 vs 2mCA):		t = -4.6, p < 0.005**	t = -1.6, p = 0.235
	MS3(0 vs 2mCA):		t = -4.7, p < 0.005**	t = 0.4, p = 0.649
	MS4(0 vs 2mCA):		t = -1.0, p = 0.325	t = 0.8, p = 0.526
	MS5(0 vs 2mCA):		t = -4.2, p < 0.005**	t = -1.5, p = 0.235

c. NREM sleep

		Coverage	Occurrence	GEV
**Age (0 vs 2mCA)**		F(1,42) = 1.9, p = 0.169	F(1,42) = 5.9, p = 0.019*	F(1,42) = 0.99, p = 0.324
**Microstate**		F(5,210) = 18.4, p < 0.005**	F(5,210) = 17.6, p < 0.005**	F(5,210) = 31.7, p < 0.005**
**Age x Microstate**		F(5,210) = 9.4, p < 0.005**	F(5,210) = 10.2, p < 0.005**	F(5,210) = 11.1, p < 0.005**
**Post hoc**	MS mean (0 vs 2mCA):			
	MS1(0 vs 2mCA):	t = 1.9 , p = 0.088	t = 1.4 , p = 0.261	t = 2.8 , p = 0.018*
	MS2(0 vs 2mCA):	t = 2.2, p = 0.060	t = 0.5, p = 0.587	t = 2.4, p = 0.033*
	MS3(0 vs 2mCA):	t = -1.7 , p = 0.118	t = -3.1 , p = 0.006*	t = -2.0 , p = 0.061
	MS4(0 vs 2mCA):	t = -5.0, p < 0.005**	t = -5.4, p < 0.005**	t = -4.9, p < 0.005**
	MS5(0 vs 2mCA):	t = 2.6, p = 0.039*	t = 0.6 , p = 0.537	t = 3.3, p = 0.006*
	MS6(0 vs 2mCA):	t = -1.6, p = 0.118	t = -3.2 , p = 0.006*	t = -1.1, p = 0.257

Changes in microstate metrics with maturation during sleep

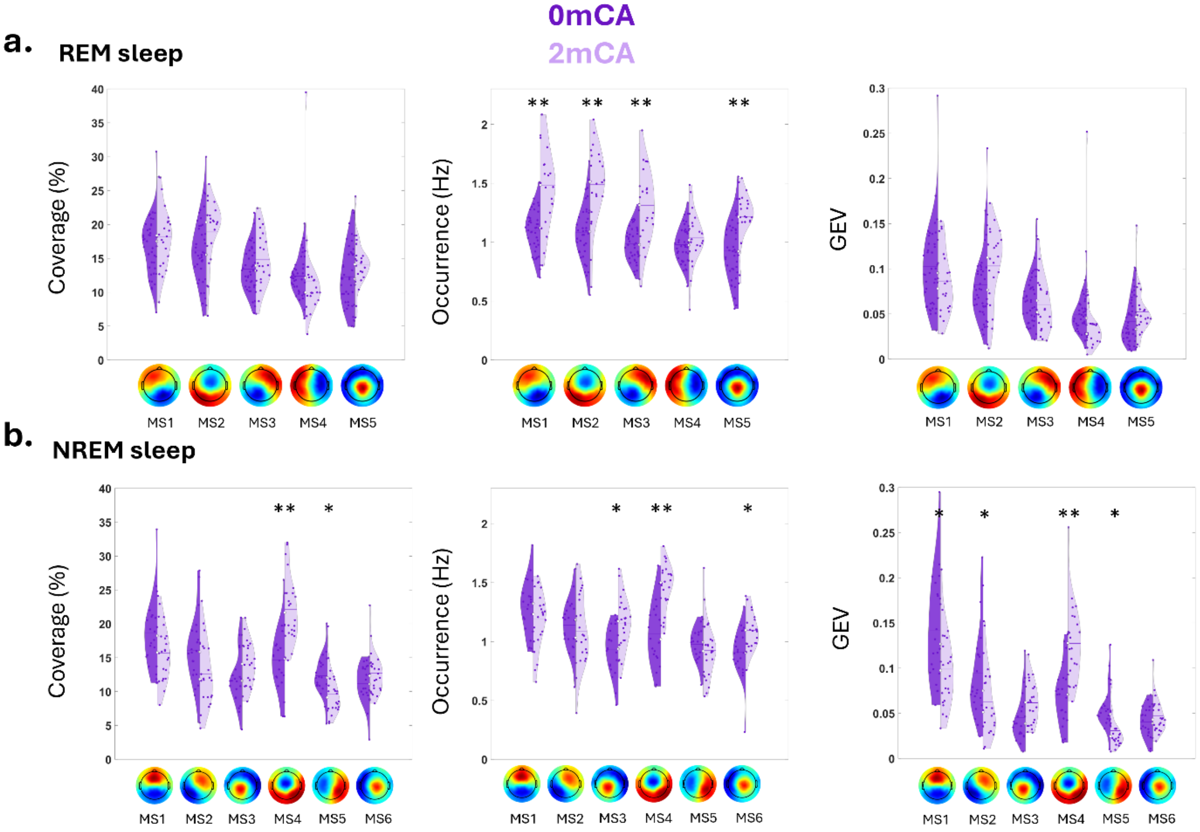

SI Figure 2. Evolution in microstate metrics (coverage, occurrence and global explained variance-GEV) between 0mCA (dark purple) and 2mCA (light purple) in preterms, during different sleep states for the **a.** five shared microstates in REM sleep; **b.** six shared microstates in NREM sleep. Asterisks represent significant differences between 0mCA and 2mCA infants (** p<0.005, *p<0.05).

3.2. Maturation of microstates transitions

Besides metrics describing individual microstates, we also explored the transitions between microstates, and quantified the transition probabilities of microstates, indicating how frequently microstates of a certain class are followed by microstates of other classes (transition probability of X⇒Y: number of times X is followed by Y divided by the number of total transitions). Since transition probabilities between microstates also depend on the base occurrence rate of each microstate, we divided (i.e., normalized) the transition probabilities by the occurrence rate of the 'destination' microstates (i.e., the microstate that the transition goes into). To assess whether each microstate has favorable microstates to transition into at each age, we compared the normalized transition probability between a pair of microstates (i.e., an initial microstate and a destination microstate) and the alternative transitions from the initial microstate using paired t-tests: the transition was considered favorable if higher than at least half of the alternative transitions. To have comparable results between the two ages and evaluate whether such transitioning patterns change with development, we focused on the shared microstates between the two age groups for each vigilance state. We corrected t-test statistics for multiple comparisons using the False Discovery Rate (FDR) approach.

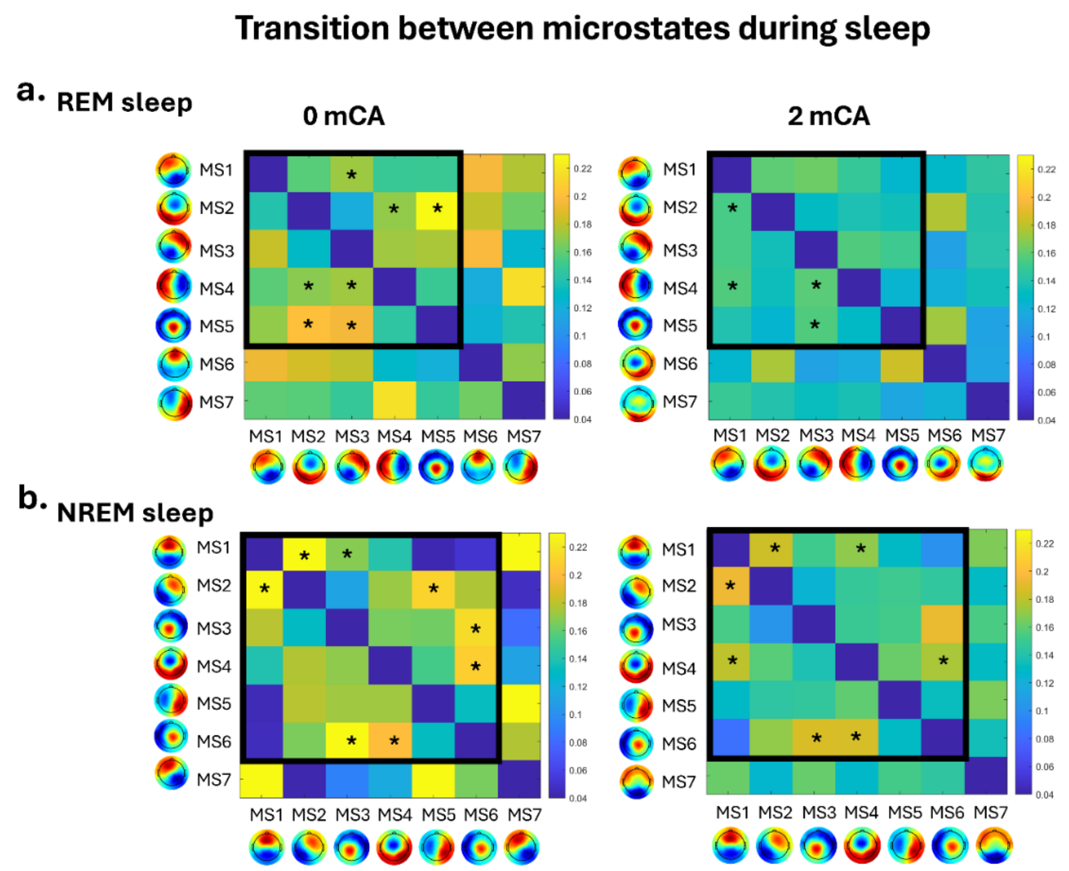

SI Figure 3. Normalized transition probabilities between different microstates at different sleep states in preterms at 0 and 2mCA (left/right column): a. REM sleep b. NREM sleep. Asterisks represent significant favorable transitions from MS X➔MSY (MS X represented in rows and MS Y represented in columns). The dark squares highlight the shared microstates between 0 and 2mCA for each vigilance state.

Comparing the normalized transition probability of pairs of microstates indicated that several microstates had significant favorable transitions, whose number varied with age and sleep state (from 4 to 8). These transitions were in part stable between 0 and 2mCA for each sleep state. For REM sleep, two of the transitions remained stable between the two ages (out of the 7/4 favorable transitions identified at 0/2mCA) (SI Figure 3.a), while for NREM sleep, five transitions remained stable (out of the 8/7 favorable transitions identified at 0/2mCA) (SI Figure 3.b).

These results indicate that at both ages, the transitions between the microstates had a non-random structure, with most microstates tending to transition into their 'favorite' microstates (one or more than one). The pattern of such transitions evolved with development between 0 and 2mCA which could suggest a reorganization of the brain functional dynamics, showing an evolution in the continuous shifting between transient network activities. Note that although the presence of non-random structure in the transition between microstates is widely accepted, test-retest reliability assessments of 'favorite' transitions have been described as poor (Antonova et al., 2022; Kleinert et al., 2023; but see Liu et l., 2020; Jun et al., 2024), limiting their interpretation for capturing interindividual differences. Future work is needed to better characterize the dynamic transitions between states, perhaps by studying the brain states in finer details beyond sleep/wake states (e.g., during or following different sensory stimulation contexts). Moreover, with the number of possible transitions between the states, studying these aspects of microstate dynamics would require a larger sample of infants for reliable statistical testing.

Antonova, E., Holding, M., Suen, H. C., Sumich, A., Maex, R., & Nehaniv, C. (2022). EEG microstates: functional significance and short-term test-retest reliability. *Neuroimage: Reports,* 2(2), 100089

Liu, J., Xu, J., Zou, G., He, Y., Zou, Q., & Gao, J. H. (2020). Reliability and individual specificity of EEG microstate characteristics. *Brain Topography,* 33, 438-449.

Kleinert, T., Koenig, T., Nash, K., & Wascher, E. (2024). On the reliability of the EEG microstate approach. *Brain topography*, 37(2), 271-286.

Jun, S., Alderson, T. H., Malone, S. M., Harper, J., Hunt, R. H., Thomas, K. M., ... & Sadaghiani, S. (2024). Rapid dynamics of electrophysiological connectome states are heritable. *Network Neuroscience*, 1-50.

4. Complementary analysis relating microstates to clinical factors

4.1. Relating microstate duration at 0mCA to GA at birth across all preterm and full-term infants in REM sleep

In a more focused analysis on the impact of GA at birth on microstates duration, we related the mean duration of microstates (averaged across all 7 microstates) at 0mCA to the group of GA at birth (GA1/GA2/GA3/Full-term) using an ANOVA and follow up t-tests. This analysis confirmed an impact of GA group on microstate duration ($F(3,46) = 3.1$, $p = 0.037$)) and posthoc tests indicated that full-term neonates have shorter microstate duration than all other preterm GA groups ($p<0.05$ for GA1 and GA3 compared to Full-terms, and $p<0.1$ for GA2 compared to full-terms).

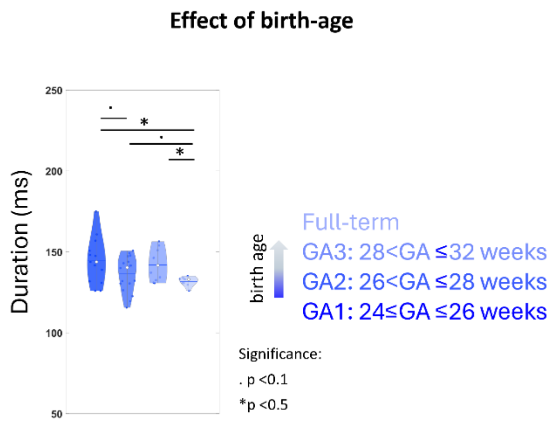

SI Figure 4. Impact of Groups of GA at birth on average MS duration in REM sleep. Asterisk and dots represent significant (p<0.05) and trend (p<0.1) differences between GA groups.

4.2. Relating microstate duration at REM sleep and clinical factors in preterms

We performed two complementary ANOVAs similar to section 2.6.3, in order to explore the relationships between microstates duration and the prematurity risk factors. Instead of considering a binary morbidity score describing non-neurological complications during the NICU period, we considered a continuous ratio variable [0:0.2:1] summarizing how many of the five factors (chronic lung disease, need for invasive mechanical ventilation lasting strictly more than 1 day, necrotizing enterocolitis, need for parenteral nutrition longer than 3 weeks, and experiencing sepsis), were present in each infant. Due to the limited number of infants, we had to consider the average duration of microstates as dependent variable (thus the microstate factor was not included in the analysis). The remaining dependent variables (GA group, MRI score, Sex, Small weight for GA) were similar to the previous analysis in 2.6.3. The following results showed no effect, in line with the results of the analysis with the binary factor.

SI Table 4. Impact of perinatal factors on microstates at 0mCA and their maturation between 0mCA and 2mCA. The relationships between microstate duration at 0mCA (n=41, left column) or the longitudinal changes in microstate duration between 0mCA and 2mCA (n=21, right column) and different prematurity risk factors were evaluated for REM sleep activity. These factors included group of GA at birth (GA1/GA2/GA3); sex; binarized risk of birth weight indicating small for gestational age; MRI Kidokoro score and continuous neonatal morbidity score summarizing non-neurological complications. At 2mCA, only main effects and interactions with adequate data points were considered. P values were corrected for multiple comparisons with FDR approach for each set of post hoc tests, and significant statistical tests are indicated with asterisk (*** p<0.0005).

	0mCA	0mCA➔2mCA
**GA group**	$F(2, 32) = 2.8$, $p = 0.075$	$F(2, 14) = 1.9$, $p = 0.185$
**MRI Score**	$F(1,31) = 0.5$, $p = 0.469$	
**Sex**	$F(1,31) = 18.7$, $p < 0.0005$***	$F(1,14) = 2.3$, $p = 0.151$
**Small Weight for GA**	$F(1,31) = 4.0$, $p = 0.089$	
**Morbidity Risk**	$F(1,31) = 1.7$, $p = 0.205$	$F(1,14) < 0.1$, $p = 0.903$
**GA group x Sex**	$F(2,31) < 0.1$, $p = 0.928$	$F(2,14) < 0.1$, $p = 0.939$

5. Comparing microstates between 0mCA and 2mCA: Impact of maturation on MS dynamics in preterms during wakefulness

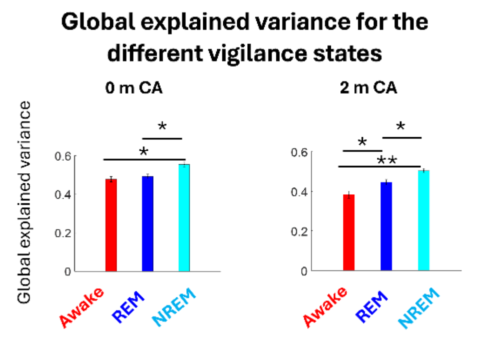

SI Figure 5. Global explained variance for the different vigilance states with 7 microstate classes. The global explained variance increased with progression in sleep depth at both ages. Significant comparisons are highlighted with asterisks (** $p<0.005$, * $p<0.05$).

SI Table 5. Maturation of microstate metrics (duration, global explained variance-GEV, coverage and occurrence) between 0mCA and 2mCA during wakefulness. Statistical comparisons were performed between 0mCA (n=17) and 2mCA (n=18): P values were corrected for multiple comparisons with FDR approach for each set of post hoc tests, and significant statistical tests are indicated with asterisk (* p<0.05, ** p<0.005).

Wakefulness

		Duration	Coverage	Occurrence	GEV
**Age (0 vs 2mCA)**		F(1,33) = 14.9, p < 0.005 **	F(1,33) = 32.0, p < 0.005**	F(1,33) = 0.4, p = 0.539	F(1,33) = 22.5, p < 0.005**
**Microstate**		F(4,132) = 0.6, p =0.647	F(4,132) = 2.6, p = 0.038*	F(4,132) = 6.7, p < 0.005**	F(4,132) = 3.8, p = 0.005*
**Age x Microstate**		F(4,132) = 1.2, p =0.305	F(4,132) = 2.5, p = 0.044*	F(4,132) = 5.0, p < 0.005**	F(4,132) = 3.2, p = 0.016*
**Post hoc**	MS mean (0 vs 2mCA):	t=3.9, p <0.005			
	MS1(0 vs 2mCA):		t=1.9, p = 0.101	t = -0.1, p = 0.911	t = 2.8, p = 0.013*
	MS2(0 vs 2mCA):		t= 2.3, p = 0.070	t = -0.1, p = 0.911	t = 3.3, p = 0.010*
	MS3(0 vs 2mCA):		t = 0.7, p = 0.500	t = 1.0, p = 0.762	t = 0.5, p = 0.643
	MS4(0 vs 2mCA):		t = -1.8, p = 0.101	t = -3.5, p = 0.005**	t = -1.3, p = 0.217
	MS5(0 vs 2mCA):		t = 3.0, p =0.030*	t = 0.4, p = 0.911	t = 3.1, p = 0.010*

Changes in microstate metrics with maturation during wakefulness

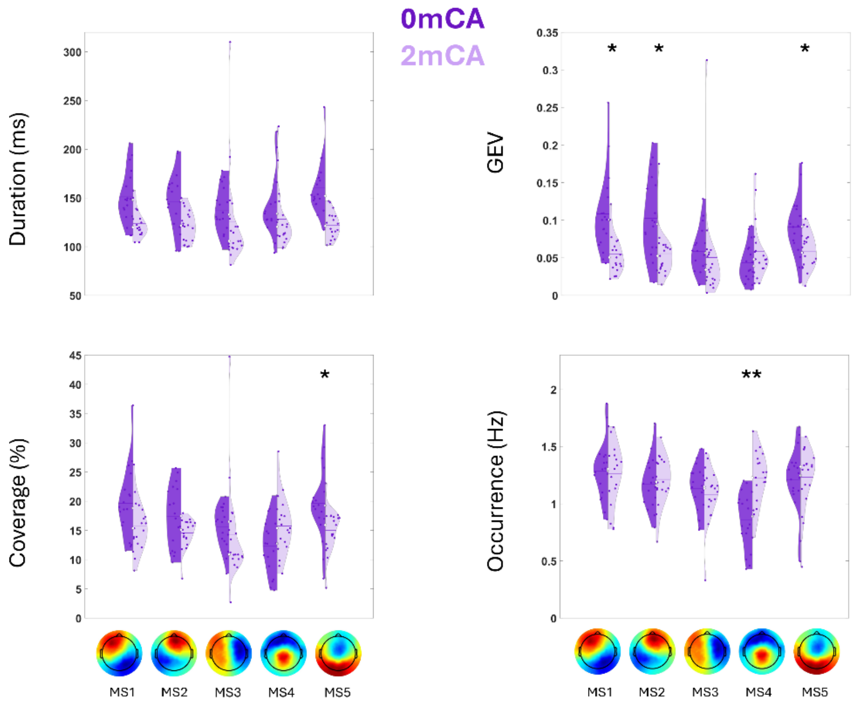

SI Figure 6. Evolution in microstate metrics (duration, global explained variance-GEV, coverage and occurrence) between 0mCA (dark purple) and 2mCA (light purple) in preterms, during wakefulness. Asterisks represent significant differences between 0mCA and 2mCA infants (** p<0.005, * p<0.05).

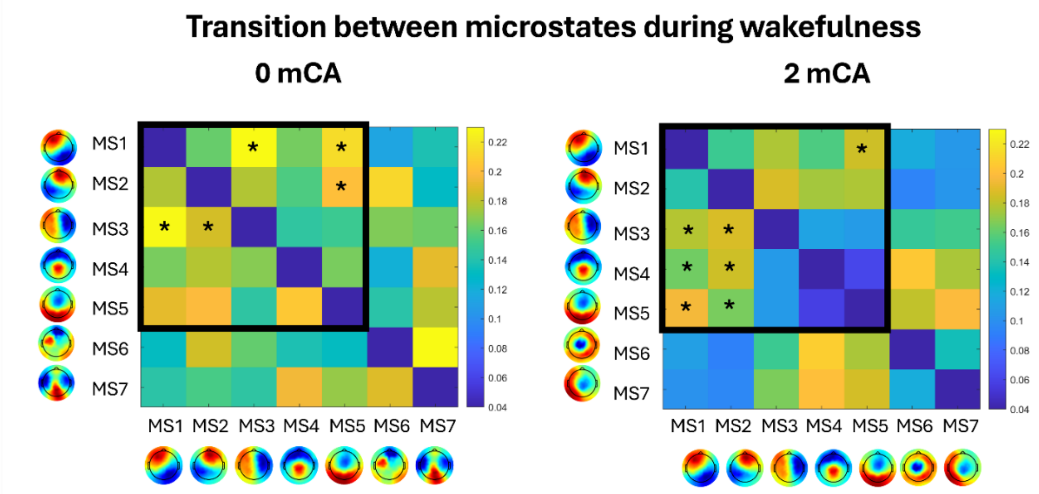

SI Figure 7. Normalized transition probabilities between different microstates during wakefulness in preterms at 0 and 2mCA (left/right column). Asterisks represent significant favorable transitions. The dark squares highlight the shared microstates between 0 and 2mCA wakefulness.

6. Summary of different microstate templates

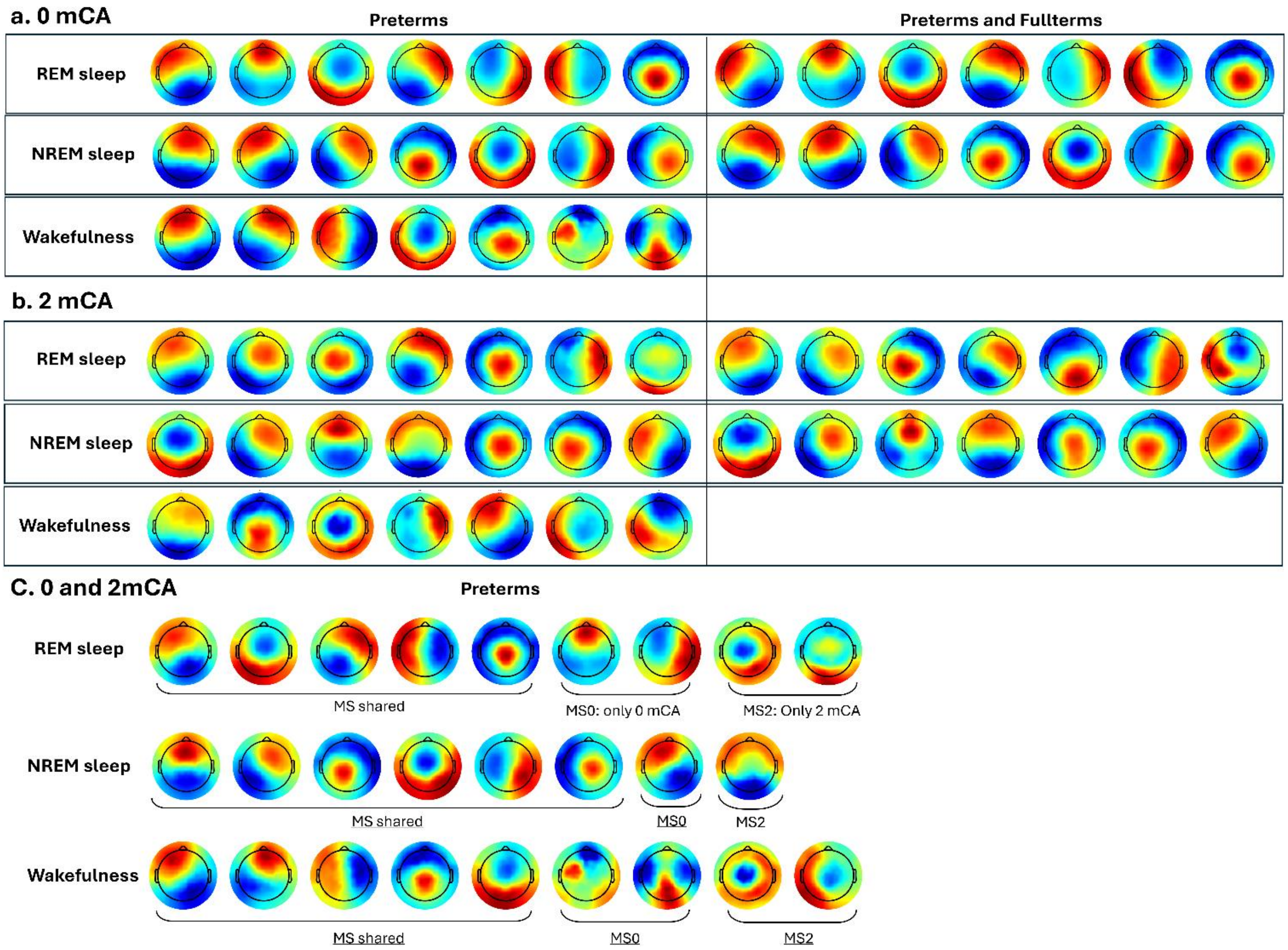

SI Figure 8. Group-level template microstates for **a.** 0mCA and **b.** 2mCA age groups across different vigilance states. Template microstates obtained considering only the preterm group are presented in the left panel, and those obtained by considering preterm and full-term infants are presented in the right panel. **c.** Group-level template microstates obtained in preterms when considering both 0mCA and 2mCA groups following the procedure described in Figure 2. These microstates involve shared patterns ("MS shared") between both age groups as well as non-shared ones (i.e. non-similar) for each age group (0mCA/2mCA → MS0/MS2).

7. Ranges of different microstate metrics

SI Table 6. Descriptive summary of microstate metrics for preterms and fullterms during REM and NREM sleep at 0 and 2mCA. Mean, ±standard deviation and [range] are indicated.

a. REM – 0mCA

	Duration(msec)				Coverage(%)				Occurrence (Hz)				GEV (ratio)			
	preterms		fullterms		preterms		fullterms		preterms		fullterms		preterms		fullterms	
	mean±std	[min max]	mean±std	[min max]	mean±std	[min max]	mean±std	[min max]	mean±std	[min max]	mean±std	[min max]	mean±std	[min max]	mean±std	[min max]
**MS1**	137±19	[104 194]	130±11	[109 144]	15±3	[8 27]	15±4	[8 21]	1.10±0.19	[0.75 1.49]	1.18±0.26	[0.75 1.55]	0.07±0.03	[0.031 0.145]	0.07±0.03	[0.02 0.12]
**MS2**	160±19	[122 221]	158±15	[141 186]	20±3	[12 29]	21±4	[16 29]	1.24±0.19	[0.87 1.65]	1.35±0.14	[1.17 1.59]	0.12±0.04	[0.063 0.235]	0.13±0.04	[0.07 0.25]
**MS3**	128±13	[101 164]	108±12	[92 127]	11±3	[4 20]	8±2	[4 12]	0.89±0.23	[0.41 1.49]	0.73±0.16	[0.52 0.94]	0.05±0.03	[0.008 0.133]	0.02±0.01	[0.01 0.05]
**MS4**	154±27	[111 221]	137±14	[121 158]	16±4	[7 28]	15±3	[11 21]	1.05±0.16	[0.69 1.46]	1.13±0.16	[0.90 1.38]	0.08±0.04	[0.026 0.183]	0.06±0.02	[0.04 0.12]
**MS5**	130±15	[100 156]	123±11	[104 148]	13±2	[8 19]	13±2	[9 20]	1.00±0.13	[0.78 1.29]	1.11±0.14	[0.89 1.37]	0.04±0.01	[0.024 0.096]	0.04±0.02	[0.02 0.09]
**MS6**	133±21	[92 194]	136±19	[113 160]	13±3	[6 24]	16±5	[10 25]	0.97±0.18	[0.60 1.42]	1.21±0.23	[0.88 1.56]	0.06±0.03	[0.026 0.183]	0.08±0.03	[0.04 0.14]
**MS7**	120±15	[87 155]	105±11	[86 118]	11±3	[3 18]	8±3	[3 12]	0.88±0.22	[0.38 1.48]	0.75±0.22	[0.40 1.03]	0.03±0.01	[0.005 0.074]	0.02±0.01	[0.01 0.03]

b. NREM – 0mCA

	Duration(msec)				Coverage(%)				Occurrence (Hz)				GEV (ratio)			
	preterms		fullterms		preterms		fullterms		preterms		fullterms		preterms		fullterms	
	mean±std	[min max]	mean±std	[min max]	mean±std	[min max]	mean±std	[min max]	mean±std	[min max]	mean±std	[min max]	mean±std	[min max]	mean±std	[min max]
**MS1**	131±24	[90 181]	141±251	[116 179]	14±5	[5 24]	17±6	[10 28]	1.06±0.25	[0.56 1.42]	1.20±0.24	[0.83 1.63]	0.09±0.05	[0.02 0.22]	0.12±0.05	[0.06 0.23]
**MS2**	153±27	[100 227]	156±41	[89 238]	19±5	[8 28]	22±9	[6 40]	1.28±0.24	[0.84 1.75]	1.36±0.30	[0.77 1.78]	0.14±0.05	[0.04 0.24]	0.17±0.11	[0.02 0.40]
**MS3**	122±21	[86 166]	118±18	[94 148]	11±3	[3 20]	12±4	[7 18]	0.94±0.18	[0.36 1.23]	1.05±0.21	[0.74 1.39]	0.05±0.02	[0.02 0.11]	0.06±0.03	[0.02 0.11]
**MS4**	130±26	[96 191]	116±18	[90 137]	11±4	[2 21]	9±4	[3 16]	0.89±0.24	[0.25 1.24]	0.79±0.28	[0.33 1.19]	0.04±0.02	[0.00 0.11]	0.03±0.02	[0.00 0.06]
**MS5**	149±20	[110 183]	134±24	[80 168]	17±5	[7 26]	13±4	[2 18]	1.15±0.27	[0.67 1.71]	0.97±0.28	[0.22 1.25]	0.08±0.04	[0.02 0.18]	0.05±0.02	[0.00 0.09]
**MS6**	125±20	[87 171]	120±12	[102 145]	11±3	[5 19]	11±2	[6 15]	0.94±0.18	[0.65 1.20]	0.97±0.16	[0.61 1.21]	0.05±0.02	[0.01 0.12]	0.05±0.02	[0.02 0.11]
**MS7**	132±19	[88 164]	125±13	[102 150]	12±3	[4 17]	12±3	[6 17]	0.97±0.18	[0.39 1.25]	0.98±0.19	[0.63 1.19]	0.05±0.02	[0.01 0.10]	0.05±0.02	[0.01 0.10]

c. REM – 2mCA

	Duration(msec)				Coverage(%)				Occurrence (Hz)				GEV (ratio)			
	preterms		fullterms		preterms		fullterms		preterms		fullterms		preterms		fullterms	
	mean±std	[min max]	mean±std	[min max]	mean±std	[min max]	mean±std	[min max]	mean±std	[min max]	mean±std	[min max]	mean±std	[min max]	mean±std	[min max]
**MS1**	132±22	[95 199]	128±19	[109 166]	21±5	[13 34]	21±6	[14 31]	1.60±0.35	[1.01 2.19]	1.66±0.30	[1.10 2.09]	0.10±0.05	[0.04 0.21]	0.10±0.06	[0.02 0.20]
**MS2**	131±27	[93 199]	115±13	[101 137]	19±6	[8 30]	13±4	[9 19]	1.47±0.29	[0.73 2.18]	1.18±0.29	[0.77 1.67]	0.08±0.03	[0.01 0.14]	0.04±0.02	[0.01 0.08]
**MS3**	120±31	[86 246]	111±11	[99 131]	16±5	[10 38]	13±2	[10 17]	1.35±0.22	[0.85 1.66]	1.17±0.13	[1.01 1.42]	0.05±0.03	[0.02 0.17]	0.03±0.01	[0.01 0.05]
**MS4**	100±15	[80 158]	102±6	[91 110]	8±3	[5 18]	9±2	[4 14]	0.88±0.25	[0.55 1.40]	0.95±0.24	[0.53 1.30]	0.03±0.02	[0.01 0.10]	0.03±0.01	[0.01 0.05]
**MS5**	117±14	[95 150]	117±10	[1023 134]	16±3	[7 21]	16±4	[9 22]	1.40±0.29	[0.76 1.86]	1.40±0.29	[0.84 1.77]	0.06±0.02	[0.01 0.09]	0.05±0.02	[0.02 0.09]
**MS6**	98±28	[73 212]	105±8	[91 118]	9±5	[3 30]	12±3	[6 17]	0.87±0.19	[0.45 1.43]	1.15±0.29	[0.63 1.63]	0.03±0.03	[0.00 0.17]	0.04±0.02	[0.01 0.08]
**MS7**	100±9	[90 124]	120±52	[91 248]	8±2	[3 13]	12±8	[7 33]	0.84±0.24	[0.33 1.26]	0.99±0.23	[0.79 1.40]	0.01±0.00	[0.00 0.04]	0.04±0.08	[0.01 0.25]

d. NREM – 2mCA

	Duration(msec)				Coverage(%)				Occurrence (Hz)				GEV (ratio)			
	preterms		fullterms		preterms		fullterms		preterms		fullterms		preterms		fullterms	
	mean±std	[min max]	mean±std	[min max]	mean±std	[min max]	mean±std	[min max]	mean±std	[min max]	mean±std	[min max]	mean±std	[min max]	mean±std	[min max]
**MS1**	128±23	[99 182]	118±20	[90 148]	15±4	[9 22]	13±5	[5 21]	1.19±0.22	[0.76 1.63]	1.10±0.38	[0.52 1.73]	0.09±0.03	[0.04 0.17]	0.09±0.05	[0.01 0.17]
**MS2**	124±27	[92 196]	101±14	[88 135]	15±5	[8 25]	10±1	[7 13]	1.23±0.26	[0.67 1.71]	0.99±0.13	[0.80 1.23]	0.07±0.03	[0.02 0.16]	0.03±0.01	[0.02 0.05]
**MS3**	109±18	[73 157]	129±39	[92 218]	9±3	[3 15]	13±6	[7 27]	0.80±0.22	[0.41 1.21]	1.02±0.16	[0.75 1.26]	0.03±0.02	[0.00 0.08]	0.07±0.05	[0.01 0.21]
**MS4**	139±23	[110 225]	142±27	[111 199]	18±4	[10 31]	19±4	[12 26]	1.31±0.20	[0.90 1.71]	1.40±0.21	[1.01 1.68]	0.11±0.04	[0.04 0.22]	0.12±0.05	[0.07 0.23]
**MS5**	119±20	[89 186]	120±18	[96 151]	13±3	[8 24]	12±3	[9 18]	1.10±0.11	[0.82 1.31]	1.03±0.17	[0.75 1.35]	0.04±0.01	[0.02 0.11]	0.04±0.02	[0.02 0.08]
**MS6**	130±15	[110 177]	122±16	[102 149]	17±3	[12 27]	15±5	[7 25]	1.37±0.25	[0.95 1.94]	1.25±0.35	[0.71 1.84]	0.06±0.02	[0.03 0.13]	0.05±0.03	[0.02 0.13]
**MS7**	104±15	[83 132]	118±23	[95 167]	10±3	[6 19]	14±5	[4 24]	1.01±0.22	[0.66 1.55]	1.21±0.28	[0.51 1.43]	0.03±0.02	[0.01 0.09]	0.06±0.04	[0.01 0.15]

SI Table 7. Descriptive summary of microstate metrics at 0mCA and 2mCA for the different vigilance states. Mean, ±standard deviation and [range] are indicated for each metric

a. REM – preterms

REM –preterms	Duration(msec)				Coverage(%)				Occurrence (Hz)				GEV (ratio)			
	0mCA		2mCA		0mCA		2mCA		0mCA		2mCA		0mCA		2mCA	
	mean±std	[min max]	mean±std	[min max]	mean±std	[min max]	mean±std	[min max]	mean±std	[min max]	mean±std	[min max]	mean±std	[min max]	mean±std	[min max]
**MS1**	151±25	[98 232]	123±15	[89 155]	16±4	[7 30]	18±4	[8 27]	1.09±0.18	[0.69 1.50]	1.47±0.35	[0.80 2.08]	0.10±0.04	[0.03 0.29]	0.08±0.03	[0.02 0.15]
**MS2**	147±25	[95 250]	126±17	[93 157]	16±4	[6 30]	18±4	[6 25]	1.08±0.23	[0.55 1.68]	1.48±0.33	[0.62 2.04]	0.08±0.04	[0.01 0.23]	0.10±0.04	[0.01 0.17]
**MS3**	135±20	[100 182]	112±15	[89 156]	13±3	[6 21]	14±4	[6 22]	0.98±0.15	[0.68 1.32]	1.31±0.31	[0.62 1.94]	0.06±0.02	[0.02 0.15]	0.06±0.03	[0.02 0.13]
**MS4**	125±17	[91 174]	108±40	[79 277]	12±2	[6 20]	11±6	[3 39]	0.97±0.15	[0.62 1.33]	1.03±0.23	[0.42 1.48]	0.04±0.01	[0.01 0.11]	0.04±0.04	[0.00 0.2]
**MS5**	130±20	[94 180]	115±22	[87 182]	12±4	[4 22]	14±3	[6 24]	0.92±0.25	[0.43 1.55]	1.21±0.21	[0.651.54]	0.04±0.02	[0.01 0.10]	0.05±0.02	[0.01 0.14]
**MS6**	150±19	[105 197]	117±27	[90 200]	15±3	[7 25]	14±5	[7 30]	1.04±0.20	[0.63 1.58]	1.22±0.27	[0.62 1.61]	0.09±0.03	[0.02 0.22]	0.06±0.04	[0.01 0.16]
**MS7**	129±14	[100 159]	109±45	[83 297]	13±3	[7 21]	7±6	[1 33]	1.01±0.18	[0.65 1.48]	0.66±0.26	[0.22 1.19]	0.05±0.02	[0.02 0.10]	0.02±0.03	[0.00 0.17]

b. NREM – preterms

NREM-preterms	Duration(msec)				Coverage(%)				Occurrence (Hz)				GEV (ratio)			
	0mCA		2mCA		0mCA		2mCA		0mCA		2mCA		0mCA		2mCA	
	mean±std	[min max]	mean±std	[min max]	mean±std	[min max]	mean±std	[min max]	mean±std	[min max]	mean±std	[min max]	mean±std	[min max]	mean±std	[min max]
**MS1**	143±27	[113 245]	131±18	[101 175]	18±5	[11 33]	15±4	[8 24]	1.29±0.23	[0.91 1.81]	1.18±0.24	[0.65 1.55]	0.14±0.05	[0.06 0.29]	0.098±0.04	[0.03 0.20
**MS2**	139±35	[88 251]	114±18	[87 162]	16±5	[5 27]	12±4	[4 23]	1.14±0.22	[0.61 1.61]	1.09±0.30	[0.39 1.65]	0.09±0.05	[0.02 0.22]	0.06±0.04	[0.010.15]
**MS3**	127±22	[95 181]	122±14	[98 161]	12.±4	[4 20]	14±3	[8 20]	0.92±0.20	[0.46 1.22]	1.15±0.23	[0.69 1.61]	0.04±0.02	[0.01 0.12]	0.06±0.02	[0.03 0.11]
**MS4**	135±18	[86 161]	151±27	[115 212]	14±4	[6 26]	22±4	[14 31]	1.07±0.27	[0.62 1.64]	1.46±0.20	[1.06 1.80]	0.06±0.03	[0.01 0.14]	0.13±0.04	[0.06 0.25]
**MS5**	125±20	[83 171]	104±12	[84 132]	12±3	[5 20]	9±3	[5 19]	0.97±0.15	[0.63 1.21]	0.92±0.25	[0.53 1.62]	0.05±0.02	[0.011 0.12]	0.03±0.02	[0.00 0.08]
**MS6**	123±16	[91 156]	115±19	[90 167]	11±3	[2 15]	12±3	[8 22]	0.91±0.21	[0.23 1.29]	1.09±0.16	[0.759 1.38]	0.04±0.02	[0.01 0.07]	0.05±0.02	[0.019 0.10]
**MS7**	128±18	[88 154]	138±45	[95 317]	15±4	[6 20]	13±5	[7 35]	1.16±0.23	[0.71 1.59]	0.93±0.13	[0.74 1.17]	0.09±0.04	[0.02 0.16]	0.06±0.05	[0.02 0.27]

c. Wakefulness – preterms

AWAKE-preterms	Duration(msec)				Coverage(%)				Occurrence (Hz)				GEV (ratio)			
	0mCA		2mCA		0mCA		2mCA		0mCA		2mCA		0mCA		2mCA	
	mean±std	[min max]	mean±std	[min max]	mean±std	[min max]	mean±std	[min max]	mean±std	[min max]	mean±std	[min max]	mean±std	[min max]	mean±std	[min max]
**MS1**	151±27	[111 206]	123±14	[104 157]	19±6	[11 36]	15±4	[8 26]	1.28±0.25	[0.86 1.87]	1.26±0.27	[0.78 1.67]	0.10±0.05	[0.04 0.25]	0.05±0.02	[0.02 0.12]
**MS2**	146±29	[95 197]	120±16	[99 149]	17±5	[9 25]	14±2	[6 17]	1.17±0.22	[0.79 1.70]	1.21±0.21	[0.67 1.58]	0.10±0.05	[0.02 0.20]	0.05±0.03	[0.01 0.17]
**MS3**	137±24	[96 177]	125±51	[81 310]	15±3	[7 20]	14±8	[2 44]	1.13±0.20	[0.77 1.48]	1.08±0.24	[0.33 1.44]	0.05±0.03	[0.01 0.12]	0.05±0.06	[0.00 0.31]
**MS4**	141±31	[93 218]	128±31	[98 223]	12±4	[4 20]	15±4	[7 28]	0.86±0.24	[0.43 1.20]	1.22±0.23	[0.70 1.63]	0.04±0.02	[0.00 0.09]	0.05±0.03	[0.01 0.16]
**MS5**	160±28	[117 243]	12±13	[101 146]	19±6	[6 33]	15±3	[5 23]	1.21±0.29	[0.49 1.67]	1.23±0.27	[0.44 1.58]	0.09±0.04	[0.01 0.17]	0.05±0.02	[0.01 0.10]
**MS6**	135±128	[70 646]	123±41	[86 270]	6±6	[1 32]	13±6	[4 34]	0.42±0.12	[0.21 0.73]	1.05±0.24	[0.49 1.53]	0.02±0.06	[0.00 0.27]	0.04±0.04	[0.00 0.17]
**MS7**	132±27	[88 180]	113±21	[89 169]	9±3	[5 17]	11±4	[4 23]	0.69±0.15	[0.37 1.12]	0.99±0.25	[0.45 1.40]	0.02±0.01	[0.00 0.05]	0.03±0.02	[0.00 0.09]
